# Electric Field‐Stimulated Autofluorescence for In Situ 3D Characterization of Polymer Defects

**DOI:** 10.1002/advs.202511290

**Published:** 2025-09-19

**Authors:** Chaolu Niu, Potao Sun, Wenxia Sima, Tao Yuan, Ming Yang, Yuhang Yang, Yuxiang Mai, Qin Deng, Yu Zhang

**Affiliations:** ^1^ State Key Laboratory of Power Transmission Equipment Technology Chongqing University Chongqing 400044 P. R. China; ^2^ School of Physics Chongqing University Chongqing 400030 P. R. China; ^3^ Analytical and Testing Center Chongqing University Chongqing 400030 P. R. China

**Keywords:** autofluorescence, electric field effect, in situ three‐dimensional characterization, microscale defect structure, stimulated emission

## Abstract

The absence of in situ detection approaches for internal defect structures in polymers (such as microscale fillers, voids, and electrical trees) has long impeded advanced materials characterization. Herein, it is demonstrate that moderate electric fields can selectively induce intense autofluorescence at defect sites within various polymers, including epoxy resins, polyethylene, silicone rubber, and polydimethylsiloxane. Such defects may evolve gradually in polymeric materials under intense electric field stress. In later stages, they can generate significant ionizing radiation, accelerating material degradation and failure. Integrating electric field‐stimulated autofluorescence with confocal laser scanning microscopy enables early detection of such defects via three‐dimensional (3D) autofluorescence imaging prior to severe irreversible damage. This approach achieves submicron 3D resolution, facilitating high‐clarity real‐time visualization of electrical tree defect dynamics. Multiscale analyses reveal that external electric fields enhance intermolecular orbital overlap, resulting in reduced HOMO–LUMO energy gaps and attenuated electrostatic potential gradients on the molecular surface. These effects promote exciton‐dominated fluorescence amplification under 552 nm excitation. Compared with existing characterization techniques, this probe‐free method offers significant advantages, including in situ and three‐dimensional imaging capabilities, thereby establishing a broadly applicable platform for analyzing microscale structures in polymers.

## Graphical abstract

Moderate electric fields induce intrinsic autofluorescence at microscale defects within diverse polymers under laser excitation. Combined with confocal microscopy, this probe‐free method enables real‐time, submicron‐resolution 3D imaging of voids, fillers, and electrical trees before irreversible damage occurs. Mechanistic insights reveal field‐driven molecular orbital shifts enhance defect‐specific fluorescence, offering a novel non‐destructive tool for in situ defect characterization.

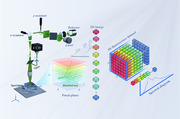

## 1 Introduction

Polymer material microstructure fundamentally dictates macroscopic performance characteristics. When subjected to high electric field stresses, the gradual evolution of intrinsic microscale defects, such as microscale fillers, voids, and electrical trees, ultimately leads to mechanical breakdown and electrical malfunction, limiting operational longevity.^[^ ^1^, ^2^, ^3^ ^]^ Although advanced characterization techniques such as atomic force microscopy (AFM)^[^ ^4^, ^5^ ^]^ and scanning electron microscopy (SEM)^[^ ^6^, ^7^ ^]^ have significantly advanced structural analysis, the in situ three‐dimensional (3D) mapping of internal microstructures—such as bubbles, impurities, electrothermal damage, and mechanical cracks—remains a persistent challenge, particularly through non‐destructive means.^[^ ^8^, ^9^, ^10^ ^]^

Existing techniques involve inherent trade‐offs. For example, while atomic force microscopy and SEM provide high‐resolution imaging, they are limited to surface characterization and cannot reveal internal structures.^[^ ^4^, ^5^, ^6^, ^7^ ^]^ Cross‐sectional slicing permits internal observation but causes irreversible damage to the sample.^[^ ^11^, ^12^ ^]^ Infrared thermography reflects temperature distribution in structurally varied regions but lacks sufficient resolution and fails to directly analyze subsurface features.^[^ ^13^, ^14^ ^]^ Three‐dimensional imaging techniques, such as ultrasound and X‐ray computed tomography (XCT), use wave or X‐ray penetration to examine internal features.^[^ ^15^, ^16^, ^17^, ^18^, ^19^ ^]^ However, these methods typically rely on projection‐based imaging techniques to highlight material density contrasts; consequently, their resolving power diminishes when the density difference between defect regions and the surrounding matrix is minimal. Furthermore, high‐energy X‐ray irradiation may induce secondary damage to the material.^[^ ^17^, ^18^, ^19^ ^]^ While phase‐contrast X‐ray imaging can resolve low‐Z materials (e.g., soft tissues), it requires specialized X‐ray sources with stringent demands on source coherence and monochromaticity.^[^ ^20^, ^21^ ^]^ Therefore, there remains a pressing need to develop 3D characterization techniques that enable in situ, non‐destructive, and high‐resolution imaging of internal microscale structures.

**Figure 1:**
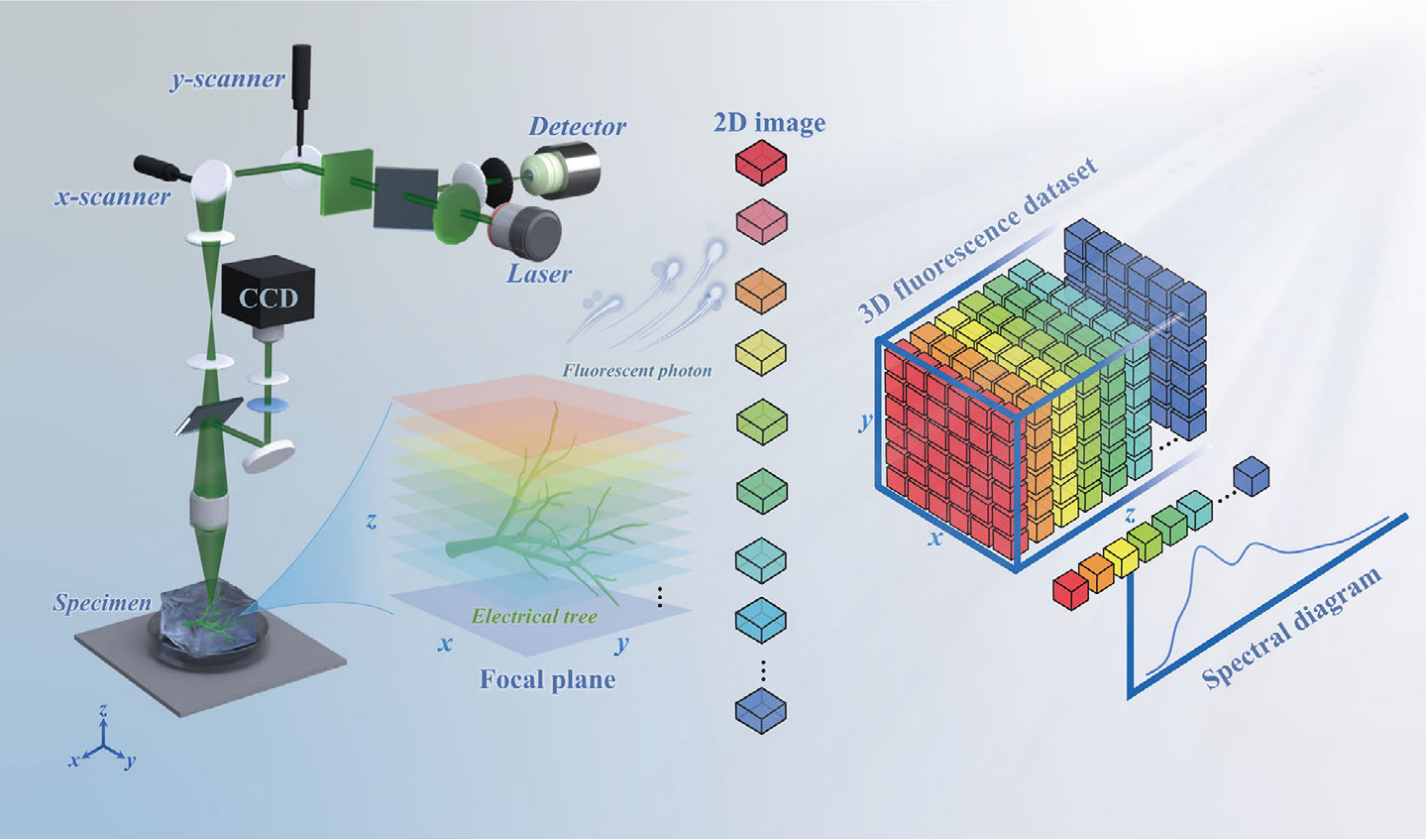
Principle of autofluorescence imaging of microscale structures in polymer materials under the influence of an electric field.

In studying the severe damage to polymers caused by strong electric fields and the detection of such damage, we have identified a novel optical phenomenon: under moderate‐intensity electric fields, non‐fluorescent polymers excited by a 552‐nm laser exhibit intrinsic autofluorescence arising from their microscopic internal defects, bubbles, and impurities. By combining electric field‐stimulated autofluorescence with confocal laser scanning microscopy (CLSM), early detection of such defects can be achieved via 3D autofluorescence imaging before the onset of severe irreversible damage. Importantly, this in situ fluorescence emission does not require exogenous probes or material modification, marking the first demonstration of in situ, probe‐free autofluorescence with cross‐polymer applicability. Mechanistic investigations indicate that electric field‐induced molecular chain scission and rearrangement at defect interfaces generate photoactive moieties capable of stimulated emission. Specifically, laser excitation elevates ground‐state molecules to excited states, which subsequently relax via spontaneous radiative decay, producing defect‐localized autofluorescence. By capturing these radiative emissions from internal defect regions using confocal laser scanning, we acquired fluorescence data across different material depths. Superimposing this depth‐resolved information enabled the reconstruction of high‐precision two‐dimensional and three‐dimensional images of microscale defect structures, as illustrated in **Figure** **1**. This approach enables accurate, in situ 3D visualization of internal microscale defects, fillers, and impurities. It holds promise as a tool for characterizing defects in polymeric materials, serving as a complement to existing SEM and XCT techniques in specific scenarios.

## 2 Results and Discussion

### 2.1 Discovery of Electric Field‐Induced Autofluorescence Effect

**Figure 2:**
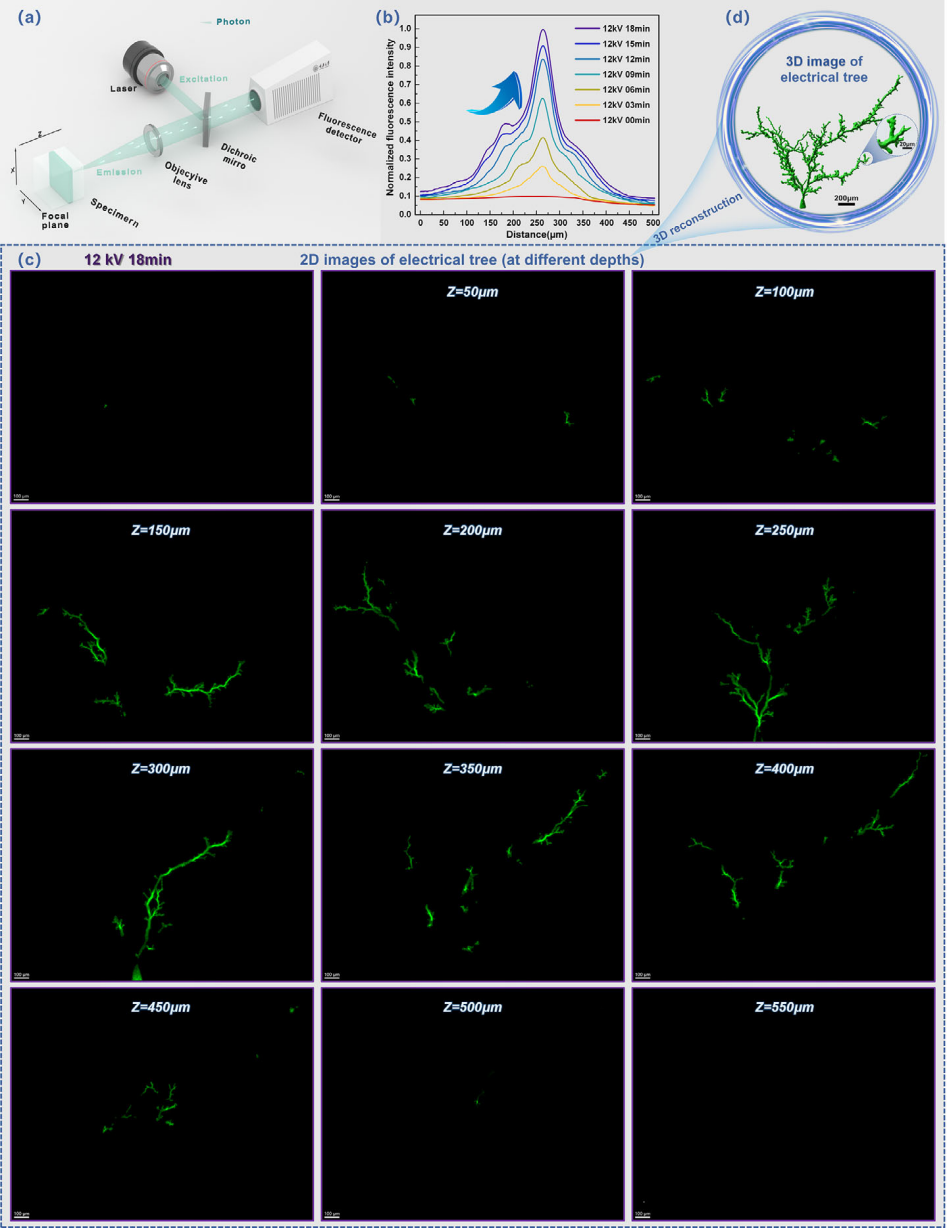
a) Principle of confocal fluorescence microscopy imaging; b) Normalized curve of two‐dimensional fluorescence intensity of electrical tree inside the epoxy resin after different times of 12 kV voltage application; c) Two‐dimensional fluorescence images of electrical tree at different focusing planes along the *z*‐axis direction (depth range of 0–550 µm) after 18 min of 12 kV voltage application; d) Three‐dimensional fluorescence image of the electrical tree and enlarged image of its tip.

In this study, we discovered that dendritic defects—referred to as electrical trees—formed in polymers under externally applied electric fields exhibit autofluorescence upon laser excitation at a specific wavelength. Unlike conventional methods that require the introduction of exogenous fluorescent probes for structural analysis, this phenomenon eliminates the need for external fluorophore labeling. Henceforth, we designate this as the electric field‐induced autofluorescence phenomenon. To validate this effect, epoxy resin was selected as the test material. A needle electrode was embedded within the sample, which was then subjected to a 12 kV alternating current voltage (50 Hz; see Figure S1, Supporting Information) until electrical trees developed inside the polymer. A 552 nm laser was applied to excite the electrical tree channels, generating fluorescent photons that enabled acquisition of a two‐dimensional fluorescence image, as illustrated in **Figure** **2**a.

As shown in the normalized fluorescence intensity curve in Figure 2b, fluorescence signal strength on the two‐dimensional plane of the electrical trees increased markedly with time under the applied electric field, suggesting that the electric field plays a critical role in initiating the autofluorescence phenomenon. To accurately obtain the three‐dimensional morphology of the electrical trees and to preliminarily demonstrate the feasibility of using this phenomenon for microscale defect characterization, we induced stimulated emission fluorescence in the defect regions using an external electric field. Specifically, a 552 nm laser was directed onto the electrical tree area after 18 min of 12 kV voltage application. At this stage, molecules within the electrical tree channels possessing fluorescent properties were excited from the ground state to the excited state. These excited molecules subsequently returned to the ground state via spontaneous emission, releasing fluorescent photons and thereby generating fluorescence signals (see Figures S3–S5, Supporting Information for further details). By capturing these photons and converting them into digital signals processed by a computer, confocal images of the corresponding emission points were generated through inversion. Point‐by‐point scanning yielded a two‐dimensional confocal image of the entire *xy* focal plane.

The sample was then scanned layer by layer along the *z*‐axis—perpendicular to the upper surface of the material—to obtain two‐dimensional fluorescence images at various depths, as indicated in Figure 1. Following 18 min of 12 kV voltage exposure, 12 sets of two‐dimensional fluorescence images covering a depth range of 0–550 µm were acquired along the *z*‐axis, as shown in Figure 2c. A three‐dimensional image of the electrical tree structure was reconstructed by dimensional elevation of the two‐dimensional datasets, as presented in Figure 2d. Using stimulated emission autofluorescence combined with confocal laser scanning microscopy (Leica Microsystems GmbH), we clearly resolved the three‐dimensional structure of the electrical tree defect. This method achieved imaging accuracies of 140 nm in the *xy* plane and 400 nm along the *z*‐axis. A comprehensive description of the confocal fluorescence imaging platform configuration is provided in Figure S5 (Supporting Information). The real‐time confocal 3D autofluorescence imaging process for polymer electrical trees is demonstrated in Movie S1 (Supporting Information).

### 2.2 Applicability of Electric Field‐Induced Autofluorescence Effect

#### 2.2.1 Autofluorescence in Different Types of Defects

**Figure 3:**
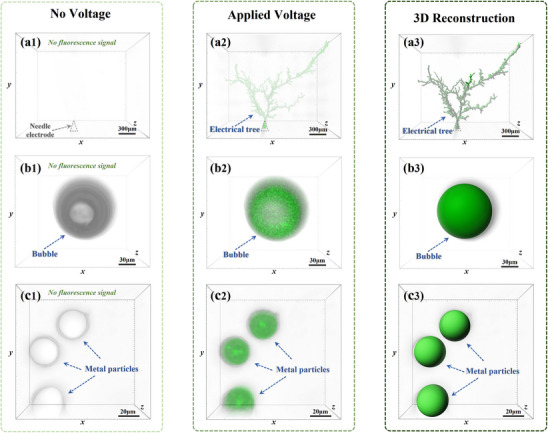
Autofluorescence imaging in epoxy resin: a1,b1,c1) Before voltage application; a2,b2,c2) After 18 min of voltage application; a3,b3,c3) Three‐dimensional reconstruction of in situ autofluorescence signals.

To investigate the applicability of the autofluorescence effect across diverse defect categories, glassy polymer epoxy resin (EP) specimens measuring 20 mm × 20 mm × 1 mm were fabricated. Needle electrodes, bubbles, and metal particles were embedded in the samples to induce electrical trees and simulate distinct defect types. Fluorescence properties before and after voltage application were analyzed using the three‐dimensional imaging method described previously; images of fluorescence overlaid with bright‐field views are presented in **Figure** **3**a–c. In the absence of an applied voltage, no electrical tree formed at the needle tip, and no autofluorescence signal was detected, as shown in Figure 3a1. Similarly, in the absence of an applied voltage, no autofluorescence signal was observed in samples containing bubbles or metal particles; however, the approximate positions of these bubbles and metal particles could be discerned under bright‐field viewing, as illustrated in Figure 3b1,c1. Using the high‐voltage power supply (needle‐plate electrode) described in detail in Figure S1 (Supporting Information), a 12 kV voltage was applied to the needle electrode in the epoxy resin sample for 18 min, resulting in the formation of an electrical tree at the needle tip. The stimulated emission corresponding to autofluorescence imaging of this electrical tree is shown in Figure 3a2. However, when the same 12 kV voltage was applied to epoxy resin samples with bubble or metal particle defects using the high‐voltage power supply (plate–plate electrode) detailed in Figure S2 (Supporting Information), no autofluorescence was observed under identical conditions. Autofluorescence signals were detected only when the applied voltage was increased to 20 kV (for bubble‐containing samples) and 28 kV (for metal particle‐containing samples), with both voltages applied for 18 min; the corresponding autofluorescence imaging results are presented in Figure 3b2,c2, respectively. It is inferred that the geometric differences between needle electrodes, bubbles, and metal particle defects lead to variations in the electric field distribution around the defects under identical voltage conditions. To explore this hypothesis, three COMSOL simulation models were constructed to analyze the electric field distribution near needle electrodes, bubbles, and metal particles, as shown in Figure S6 (Supporting Information). The simulations revealed that when voltages of 12, 20, and 28 kV were applied, the maximum local electric field strengths near the defects were 171.26, 143.17, and 144.31 kV mm^−1^, respectively. The average electric field strengths in the defect‐free regions of the polymer samples were 12, 20, and 28 kV mm^−1^, corresponding to moderate field levels. Notably, the local field strengths near the defects all exceeded 100 kV mm^−1^, indicating that autofluorescence is triggered when the internal electric field strength surpasses a specific threshold. These findings demonstrate that electric field‐induced autofluorescence can occur not only in electrical trees but also in other types of defects, providing preliminary evidence that this phenomenon can be used in combination with confocal microscopy to visualize the three‐dimensional morphology of microscale defects.

For precise geometric analysis, an automatic path planning algorithm (see Figures S7–S10, Supporting Information for details) was employed to reconstruct in situ autofluorescence signals into three‐dimensional morphologies, with results shown in Figure 3a3–c3. Taking the electrical tree defect as an example, this reconstruction effectively eliminated background noise and enabled high‐fidelity fitting of the electrical tree structure. Details of the three‐dimensional reconstruction process in epoxy resin are provided in Figures S7–S10 (Supporting Information), while Movie S2 (Supporting Information) dynamically demonstrates the reconstruction and structural evolution of the electrical tree in epoxy resin.

#### 2.2.2 Autofluorescence in Different Types of Polymer Materials

**Figure 4:**
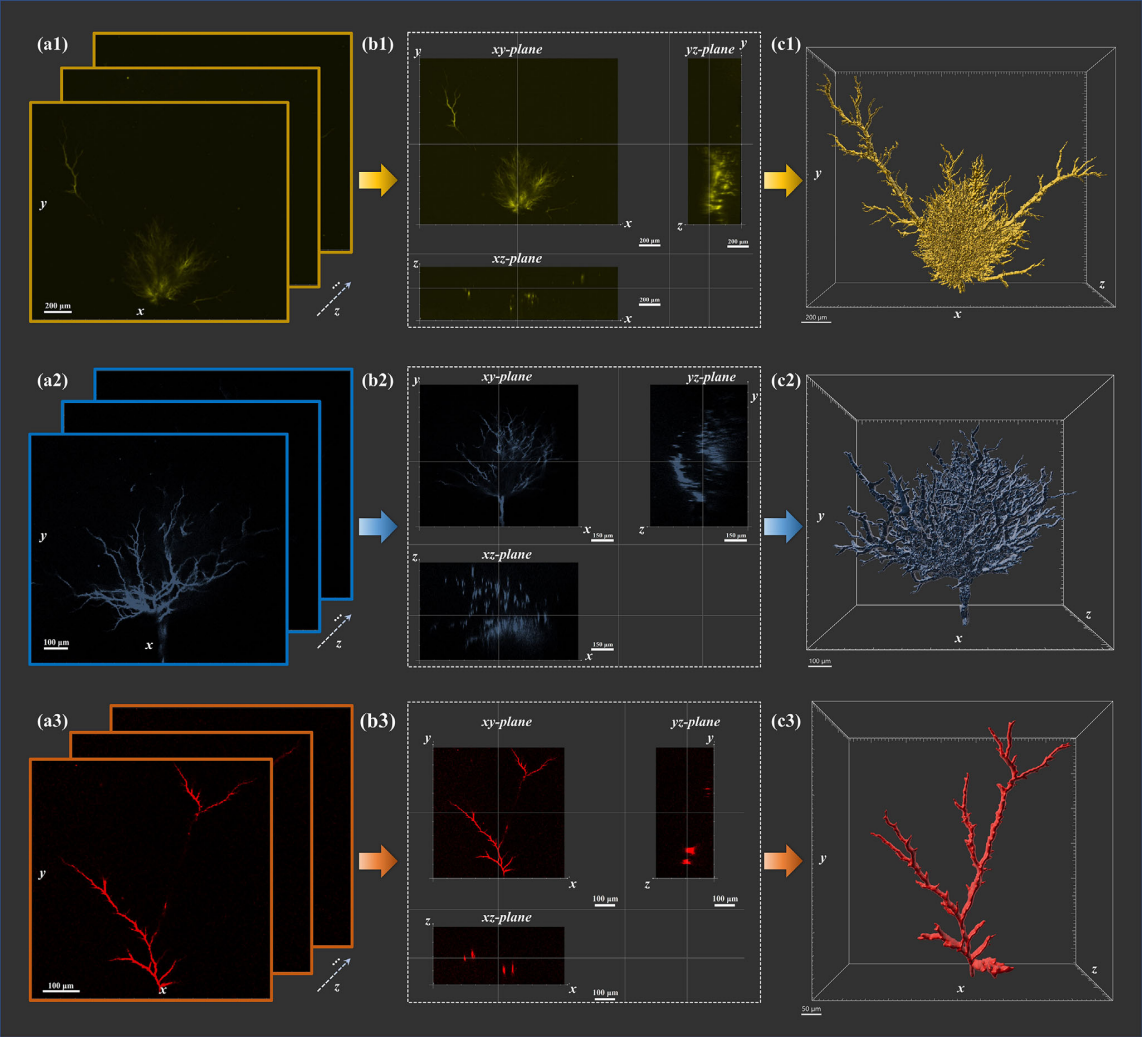
Electrical tree characterization in XLPE, RTV, and PDMS: a1–a3) Two‐dimensional confocal images of electrical trees in the *xy* focal plane at different depths; b1–b3) Original autofluorescence information at different cross‐sections (*xy*, *yz*, and *xz* planes); c1–c3) Stereoscopic morphology after three‐dimensional reconstruction.

To further evaluate the applicability of the autofluorescence effect across distinct polymer categories, representative rubbery polymers—specifically cross‐linked polyethylene (XLPE), room‐temperature vulcanized silicone rubber (RTV), and polydimethylsiloxane (PDMS)—were deliberately chosen for this study. Samples with dimensions of 20 mm × 20 mm × 1 mm were fabricated, and needle electrodes were embedded in each to generate electrical tree defects. The autofluorescence characteristics before and after voltage application were analyzed using the aforementioned three‐dimensional imaging method, with the resulting fluorescence images presented in **Figure** **4**a–c. Prior to voltage application, no electrical trees formed at the needle tips in any of the three materials, and no fluorescence signals were observed. Owing to differences in density, dielectric constant, and molecular structure, the threshold voltages required to induce electrical tree formation varied: 9 kV for XLPE, 8 kV for RTV, and 7 kV for PDMS. Using the power supply detailed in Figure S1 (Supporting Information), needle electrodes embedded in XLPE, RTV, and PDMS samples were each subjected to these voltages for 18 min, resulting in electrical tree formation at the tip of each needle electrode. By controlling the sample stage to perform layer‐by‐layer scanning along the *z*‐axis from top to bottom across samples of different materials, two‐dimensional confocal images of internal electrical trees within the *xy* focal plane at various depths were acquired for the three polymer materials, as shown in Figure 4a1–a3. These two‐dimensional autofluorescence images were subsequently constructed into three‐dimensional images to visualize the electrical tree structure. Original autofluorescence information of the electrical trees was obtained across different cross‐sections (*xy*, *yz*, and *xz* planes) of the XLPE, RTV, and PDMS samples, as illustrated in Figure 4b1–b3. For more precise spatial geometric analysis of the three‐dimensional morphology of electrical trees, three‐dimensional spatial reconstruction was performed using in situ autofluorescence generated by the electrical trees. The three‐dimensional reconstruction results of electrical tree defects in different materials are presented in Figure 4c1–c3, and the dynamic visualization of the stereoscopic morphology of the reconstructed electrical trees is provided in Movie S3 (Supporting Information). These results confirm that electric field‐induced autofluorescence is not limited to epoxy resins but also exists in other polymer types. Thus, this phenomenon can be combined with confocal microscopy to effectively characterize three‐dimensional microstructures—including electrical trees and other defects—and is applicable to both glassy polymer systems (epoxy resin) and various rubbery polymer systems (XLPE, RTV, PDMS).

### 2.3 Three‐Dimensional Dynamic Evolution of Electrical Trees

**Figure 5:**
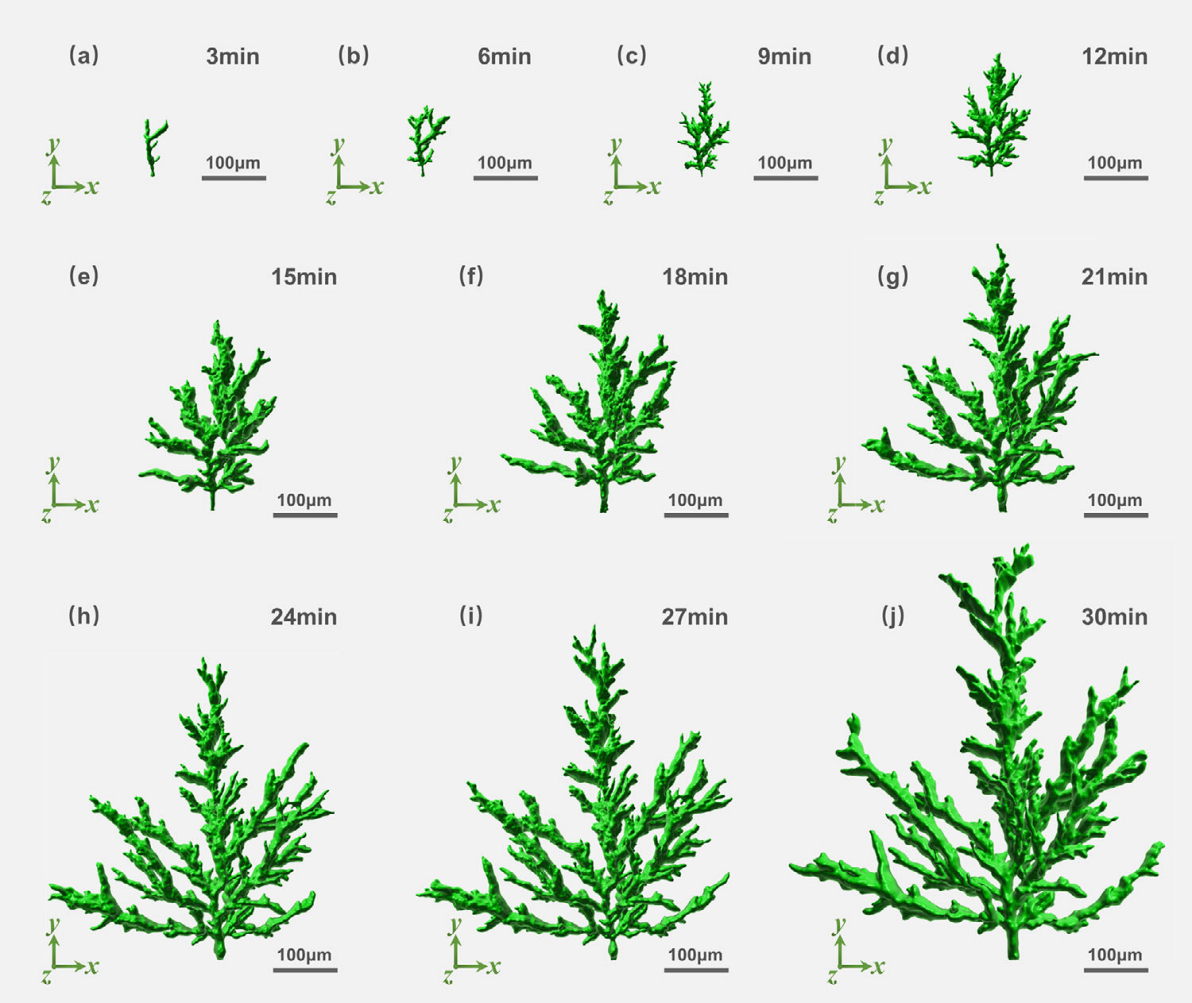
Three‐dimensional morphology and dynamic development process of electrical trees at different voltage application times: a) 3 min, b) 6 min, c) 9 min, d) 12 min, e) 15 min, f) 18 min, g) 21 min, h) 24 min, i) 27 min, j) 30 min.

For an extended period, the three‐dimensional (3D) dynamic evolution of electrical trees under the influence of electric fields has been considered unobservable. To address this limitation, we employed the electric field‐induced autofluorescence effect in conjunction with 3D reconstruction techniques to capture the real‐time morphological development of electrical trees within polymeric materials. Epoxy resin sheet samples measuring 20 mm × 20 mm × 1 mm were selected, and needle electrodes were embedded in each. Upon applying a 12 kV voltage to the needle electrodes, electrical trees were successfully initiated within the epoxy resin. In situ autofluorescence signals from the electrical trees were acquired using a two‐photon confocal fluorescence microscopy system. Subsequently, 3D reconstruction was performed to visually represent the spatial development of the electrical trees. Dynamic monitoring was conducted at 3‐min intervals for a total of 30 min, capturing the evolution of the electrical tree morphology, as depicted in **Figure** **5**a–j. As shown in Figure 5a, at 3 min under 12 kV, the electrical tree extended to dimensions of 44.11 µm along the *x*‐axis, 91.92 µm along the *y*‐axis, and 36.67 µm along the *z*‐axis. By 30 min, as shown in Figure 5j, the tree had grown to 574.35 µm in *x*, 673.94 µm in *y*, and 365.13 µm in *z*, representing increases of 1302.09%, 733.18%, and 995.72%, respectively, compared to the 3‐min values. These data indicate that the growth rate of the electrical tree accelerates significantly in all directions as the duration of voltage application increases. Furthermore, the spatial volume occupied by the electrical trees expanded markedly, resulting in an increased number of branching structures. The in situ 3D autofluorescence imaging method introduced in this study offers high‐resolution spatial insights into the micro‐ to mesoscopic structural evolution of electrical tree defects. This capability enables clearer differentiation of discharge behavior during various stages of electrical tree development, offering critical diagnostic potential for polymer insulation degradation. In addition, the effects of varying needle electrode placement (anterior/posterior positions) and quantity on electrical tree morphology are systematically documented in Figures S11 and S12 (Supporting Information).

### 2.4 Quantitative Analysis of Electric Field‐Induced Autofluorescence

This study focused on epoxy resin to quantitatively examine its fluorescence characteristics following exposure to a 12 kV electric field for various durations. Fluorescence lifetime imaging was utilized to verify the contributions of electrically induced components to autofluorescence within the electrical tree regions. Subsequently, circular dichroism (CD), UV–vis–NIR absorption spectroscopy, and thermogravimetric‐infrared analysis were performed to investigate the compositional changes induced by electric field exposure over time in the affected regions.

#### 2.4.1 Fluorescence Lifetime Analysis in Electrical Tree Regions

**Figure 6:**
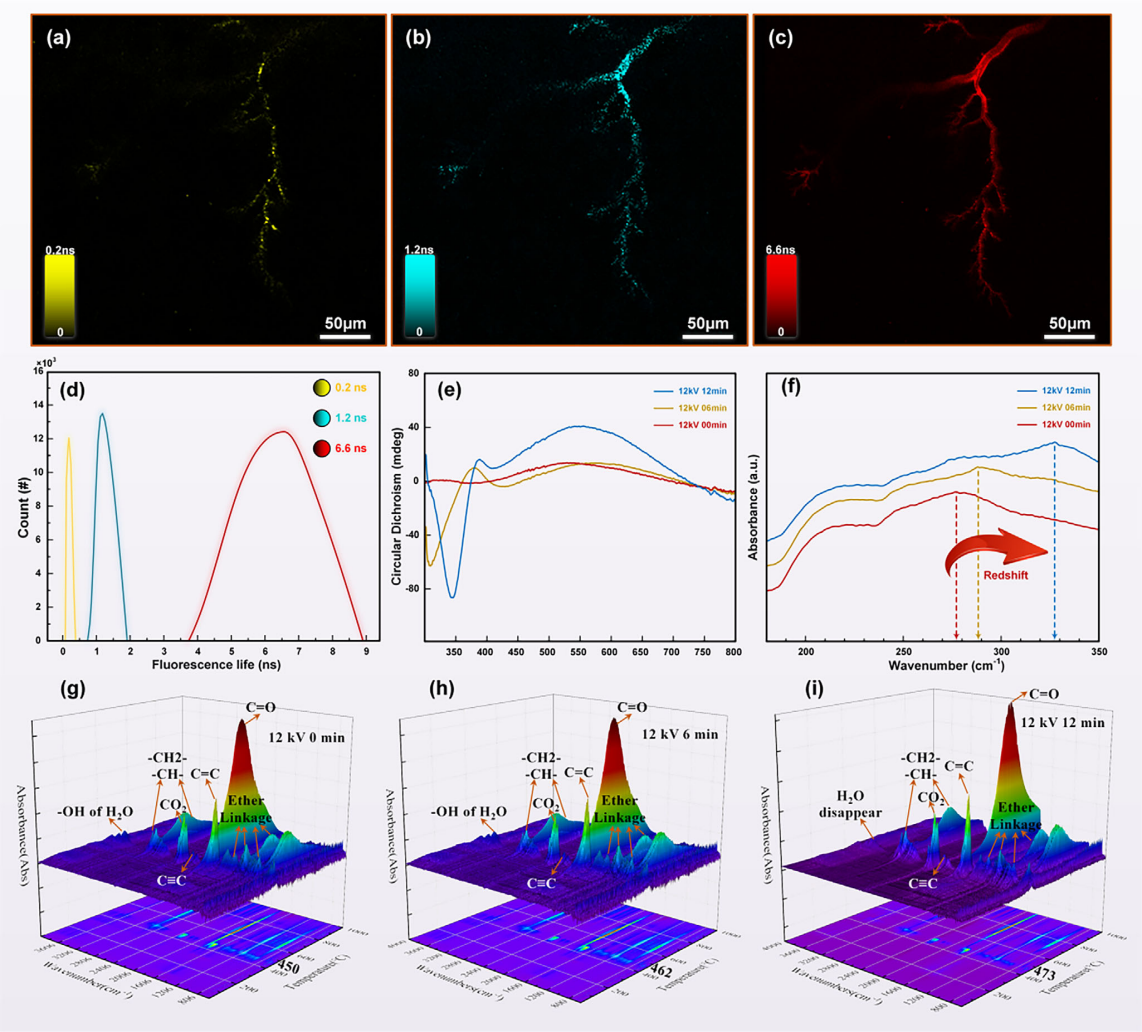
a–c) Fluorescence lifetime decomposition of electrical trees, and d) Gaussian distribution of fluorescence lifetimes. e) Circular dichroism spectroscopy, f) UV–vis–NIR absorption spectroscopy, and g–i) thermogravimetric‐infrared spectroscopy analysis of electrical trees under varying durations of 12 kV voltage application.

To elucidate the mechanism underlying stimulated autofluorescence in the electrical tree regions of epoxy resin, it is first necessary to classify and identify the fluorescent species within the channels. Fluorescence lifetime, an inherent property of fluorophores, defines the time a molecule remains in the excited state before returning to the ground state via emission. Since each fluorescent molecule exhibits a distinct lifetime, different fluorescent groups can be distinguished based on their emission dynamics,^[^ ^22^ ^]^ thus enabling identification of the luminescent species involved. Analysis of the autofluorescence signals in the electrical tree regions revealed that fluorescence lifetimes ranged from 0.1 to 8.9 ns, as shown in **Figure** **6**a–d. By fitting the decay curves of fluorescence intensity at each pixel, the fluorescence lifetime values corresponding to decay components from different fluorescent groups were extracted. The results revealed three distinct lifetime populations within the electrical tree regions. Based on the Gaussian distribution shown in Figure 6d, the average lifetimes of the three fluorescent species were determined to be 0.2, 1.2, and 6.6 ns, respectively. Notably, the fluorescent component with the longest lifetime emitted the highest number of photons, suggesting that it was either the most abundant or possessed functional groups more susceptible to electric field‐induced autofluorescence. These findings imply that at least three distinct types of fluorescent substances are involved in the autofluorescence phenomenon of electrical trees within epoxy resin. Further identification and differentiation of these substances are essential to gain comprehensive chemical and physical insights into the autofluorescence mechanism.

#### 2.4.2 Circular Dichroism Spectroscopy Analysis

To further explore molecular‐level changes induced by electric fields in the regions containing electrical trees, circular dichroism (CD) spectroscopy was employed to investigate structural alterations in the epoxy resin. As shown in the CD spectrum in Figure 6e, the epoxy resin sample that was not exposed to an electric field exhibited negligible circular dichroism, with intensity values close to zero. However, upon exposure to the electric field, CD signals became evident and intensified progressively with increased exposure duration. After 12 min under a 12 kV electric field, the circular dichroism intensity at 347 nm reached −88.9 mdeg, indicating a pronounced chiroptical response in the material. In the visible spectrum range of 320–800 nm, certain small organic molecules containing chromophores may exhibit CD activity, primarily arising from variations in electron distribution due to electronic transitions. These results suggest that electric fields induce molecular chain scission within the polymer, generating numerous organic small molecules with conjugated structures. The emergence of these conjugated systems enhances optical absorption and contributes to the observed increase in circular dichroism within the visible region. This finding provides preliminary evidence that electric field exposure alters polymer structure at the molecular level, contributing to the emergence of autofluorescent properties.

#### 2.4.3 UV–vis–NIR Absorption Spectroscopy Analysis

As illustrated in the UV–vis–NIR absorption spectrum (Figure 6f), a series of characteristic absorption peaks within the wavelength range of 180–350 nm confirms the presence of benzene rings in the material. Notably, strong absorption peaks were observed between 275–335 nm, indicating π→π* transitions involving at least 3–5 conjugated units. With increasing voltage application time, the influence of π electrons participating in π* transitions became more pronounced. Simultaneously, the conjugation within molecular chains was enhanced under the applied electric field. This enhanced conjugation—particularly between carbonyl (C═O) and carboxyl groups and olefinic bonds—reduces the energy of the π* orbitals. As a result, both π→π* and *n*→π* transition peaks shift toward longer wavelengths, accompanied by increased absorption intensity. These findings suggest that electric field‐induced scission of epoxy resin molecular chains leads to the generation of small conjugated molecules, which are likely responsible for the observed stimulated radiative fluorescence. The breakdown of polymer molecules under electric‐field‐induced thermal stress shortens the effective conjugation length, resulting in a redshift in the autofluorescence absorption spectrum.

#### 2.4.4 Thermogravimetric‐Infrared Spectroscopy Analysis

Thermogravimetric(TG)‐infrared analyses were conducted on epoxy resin samples subjected to varying durations of 12 kV voltage application to investigate changes in material composition during different stages of electrical treeing. The time interval between the termination of high‐voltage application (12 kV) and the initiation of TG‐infrared analysis was strictly controlled to within 30 min for all samples. The corresponding results are presented in Figure 6g–i. As shown in Figure 6g, no significant characteristic absorption peaks appear in the infrared spectrum below 450 °C, indicating that pure epoxy resin exhibits good thermal stability and does not undergo severe electrothermal decomposition below this temperature. Figure 6g–i reveals that the molecular chains of the epoxy resin begin to decompose significantly at temperatures of 450, 462, and 473 °C, respectively. In the infrared region from 4000–3400 cm^−1^, absorption peaks correspond to the stretching vibration of –OH groups, indicative of water (H_2_O) production. This is primarily due to the electrothermal decomposition of epoxy resin molecular chains, suggesting that H_2_O generation and escape accompany the decomposition process. Furthermore, as the duration of voltage application increases, the amount of H_2_O generated during subsequent electrothermal decomposition decreases, and the –OH characteristic peak becomes less prominent, as illustrated in Figure 6g–i. In the region from 2900 to 3000 cm^−1^, the methyl (–CH_2_–) and methine (–CH–) groups in the epoxy resin show C–H stretching vibration absorption peaks. The absorption peak near 2358 cm^−1^ is primarily attributed to CO_2_, suggesting that electrothermal decomposition at this stage produces unsaturated olefins and alkynes, which subsequently oxidize in air to form CO_2_ and aldehydes. Between 1000 and 1355 cm^−1^, multiple characteristic peaks corresponding to ether bonds are observed. The peak near 1650.12 cm^−1^ is assigned to the C═C stretching vibration, the peak around 1732.31 cm^−1^ corresponds to C═O stretching, and the peak at ≈2181 cm^−1^ is attributed to C≡C stretching. The intensities of these three peaks increase with longer 12 kV voltage exposure, likely due to the accumulation of organic small molecules rich in C═C and C═O bonds, which are produced through electrothermal decomposition induced by electrical treeing within the epoxy resin. When the temperature exceeds 800 °C, the characteristic absorption peaks in the TG‐IR spectra decline significantly and then stabilize, indicating that the formation of electrothermal decomposition products has reached a steady state. This suggests that as the temperature continues to rise, no new decomposition products are formed, and the reaction enters a thermodynamic plateau phase. In future work, molecular dynamics simulations based on the reactive force field (ReaxFF) will be used to quantitatively investigate fluorescent substances formed in internal electrical tree defect regions under the coupled effects of electricity and heat. Additional details concerning the Raman spectroscopy, Fourier transform infrared spectroscopy, and thermogravimetric analyses performed as supplementary studies are provided in Figures S13–S15 (Supporting Information).

### 2.5 Molecular Dynamics Simulation

In this section, we investigate changes in the physical and chemical properties of epoxy resin before and after electrothermal decomposition under electric field influence, from the perspective of condensed matter systems. Building upon the previous experimental analyses, a ReaxFF molecular dynamics model was developed to examine the effects of electrothermal coupling stress on epoxy resin during the development of electrical trees. This approach reveals the structural transformation mechanisms and elucidates the role of organic fluorescent small molecules in the observed autofluorescence phenomenon.

#### 2.5.1 ReaxFF Molecular Dynamics Simulation of the Epoxy Resin System

**Figure 7:**
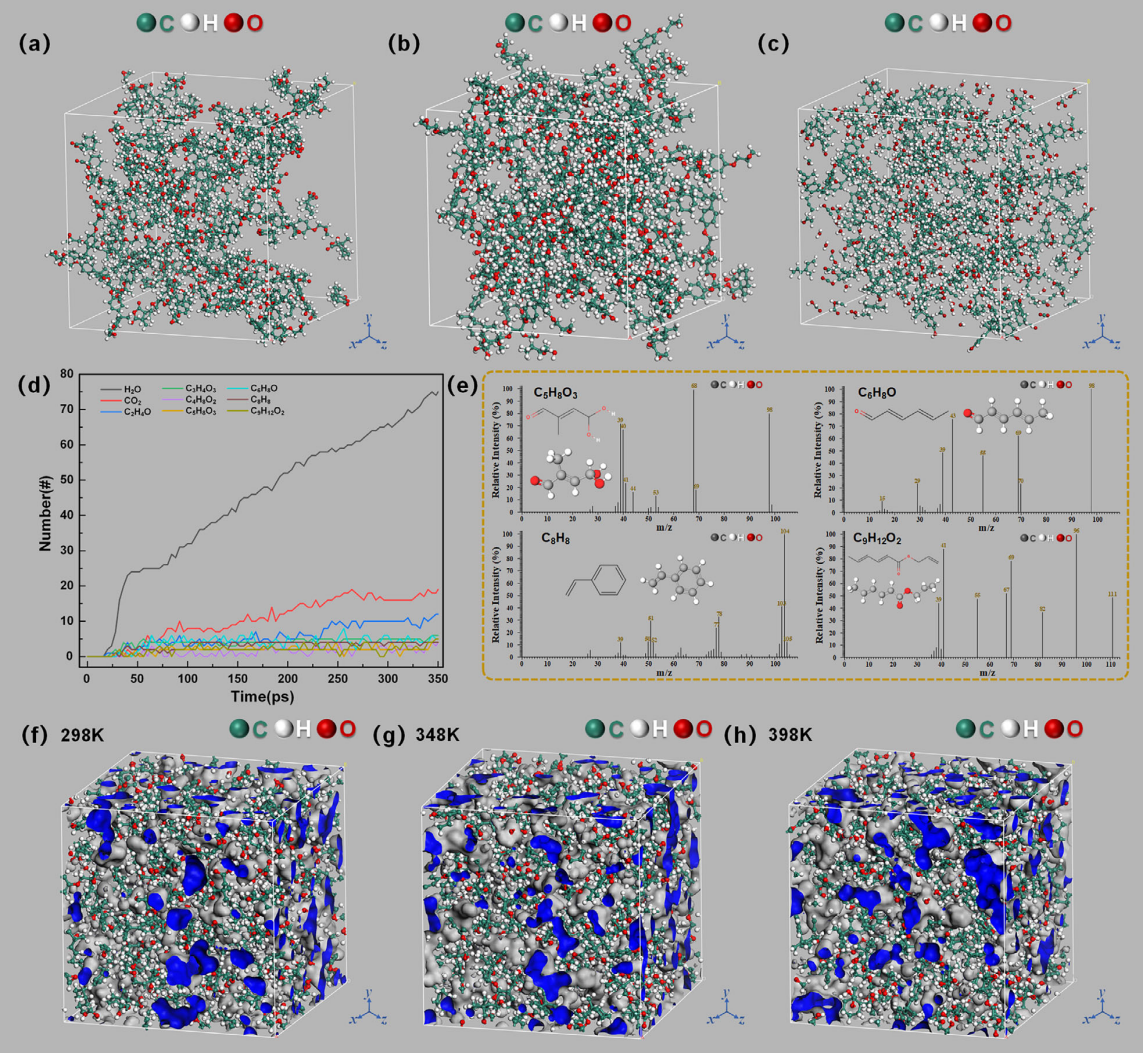
a) Initial state of the epoxy resin system before crosslinking; b) Final state of the epoxy resin system after crosslinking and structural optimization; c) Final state of the epoxy resin system following electrothermal decomposition; d) Changes in the newly generated products of the epoxy resin system during electrothermal decomposition; e) Mass spectrum and molecular structure of solid products formed by electrothermal decomposition of epoxy resin; f) free volume distribution of the epoxy resin system at 298 K; g) free volume distribution at 348 K; h) free volume distribution at 398 K.

Bisphenol A diglycidyl ether (DGEBA) was selected as the epoxy resin matrix, and methyl hexahydrophthalic anhydride (MHHPA) was used as the curing agent to construct the simulation system. The epoxy equivalent of DGEBA is ≈196 (range: 185–208), and it exists in a liquid state at room temperature with a polymerization degree (*n*) of 0 or 1.^[^ ^23^ ^]^ The molecular structures and crosslinking reaction mechanisms of DGEBA and MHHPA are illustrated in Figures S16–S19 (Supporting Information). A molecular dynamics model of the crosslinked epoxy system was constructed via a Perl script, as shown in Figure S20 (Supporting Information). The initial state of the epoxy resin system prior to crosslinking is presented in **Figure** **7**a, while the crosslinked and optimized configuration is shown in Figure 7b. In the ReaxFF molecular dynamics simulation, temperature primarily influences the reaction rate and extent of decomposition but does not alter the reaction pathway or final products. Therefore, to improve computational efficiency, an electrothermal decomposition simulation was performed over 350 ps at 2000 K, following a pre‐equilibration phase of 20 ps. The simulation was conducted using the NVT ensemble, with a time step of 0.1 fs. An external electric field was introduced by applying a force *F* = *qE* to each atom, where *E* represents electric field strength.

Referring to the threshold electric field values that induce autofluorescence in polymers (Figure S6, Supporting Information), an electric field strength of *E* = 100 kV mm^−1^ was applied in the *z*‐axis direction of the epoxy resin system (Figure 7). The system configuration under both ReaxFF and electric field influence is depicted in Figure 7c. The relationship between the number of newly generated molecular species and reaction time under combined thermal and electrical stress is shown in Figure 7d. The appearance of gas‐phase products such as CO_2_, H_2_O, and C_2_H_4_O marks the onset of decomposition. With continued decomposition, liquid and solid molecular products such as C_3_H_4_O_3_, C_4_H_8_O_2_, C_5_H_8_O_3_,C_6_H_8_O, C_8_H_8_, and C_9_H_12_O_2_ appeared, suggesting that high‐energy discharge processes had occurred. Coupled with gas chromatography‐mass spectrometry (GC‐MS) analysis, several representative small‐molecule solid products were further identified. Their molecular formulas, ball‐and‐stick models, and corresponding mass spectra are presented in Figure 7e. After electrothermal decomposition, the system exhibited an increased presence of small organic molecules containing carbon–carbon double bonds (C═C) and carbon–oxygen double bonds (C═O), which reduced intermolecular distances and enhanced π–π* and *n*–π* conjugation interactions.^[^ ^24^, ^25^ ^]^ Notably, the aggregation of solid‐phase molecules such as C_5_H_8_O_3_, C_6_H_8_O, C_8_H_8_, and C_9_H_12_O_2_ increased steric hindrance and restricted the non‐radiative decay of excited electrons, thereby promoting stimulated autofluorescence. The observed autofluorescence effect can thus be attributed to the widespread presence of conjugated π‐electron systems (e.g., C═C, C═O) and their associated *n*–π* and π–π* interactions in the aggregated phase following electrothermal decomposition.^[^ ^26^, ^27^ ^]^ These π‐conjugated units (including heavy bonds and functional groups) tend to cluster under electrothermal coupling conditions. When the interatomic distances fall below the sum of their van der Waals radii, effective electronic interactions occur, leading to extended electron delocalization.^[^ ^28^, ^29^ ^]^ Consequently, when these clustered chromophores become sufficiently rigid, they can be excited and emit significant autofluorescence as they transition from the excited to ground state.

#### 2.5.2 Effect of Temperature on the Free Volume of the Epoxy Resin System

The fractional free volume (FFV) is a critical parameter reflecting the morphology and thermomechanical stability of polymer systems. It is defined by the equation:

(1) $$\mathrm{FFV}=V_{f}/\left(V_{f}+V_{o}\right)\times 100\%$$

where *V*_f_ represents the free volume and *V*_o_ denotes the occupied volume

Understanding how temperature affects the FFV of a crosslinked epoxy resin system provides a foundational basis for elucidating the molecular‐level mechanisms by which thermal effects influence the development of electrical trees. Using the previously established molecular dynamics model of the crosslinked epoxy resin system (Figure 7b), a 1000 ps free volume simulation was performed in the Forcite module. A pre‐equilibration phase of 20 ps was followed by a production run in the NVT ensemble using the COMPASS II force field with a time step of 0.2 fs. The Connolly surface method was employed to compute the free volume at different ambient temperatures.

The free volume distributions at 298, 348, and 398 K are presented in Figure 7f–h. The corresponding free volume proportions were 16.36%, 17.88%, and 19.31%, respectively. This increasing trend indicates a positive correlation between temperature and free volume in the epoxy resin molecular system. As temperature rises, the thermal motion energy of the molecular chains increases. When this energy surpasses the internal rotational barriers, segmental motions are activated, resulting in increased free volume.^[^ ^30^ ^]^ Moreover, the expansion and coalescence of free volume regions may blur the boundaries between distinct aggregation domains, thereby reducing the thermomechanical stability of the epoxy resin. This facilitates molecular chain scission by charge carriers at lower energy thresholds, ultimately promoting the initiation and propagation of electrical trees. These findings offer molecular‐level insight into the enhancing effect of elevated temperatures on electrical tree development in epoxy resins.

### 2.6 Quantum Chemical Analysis

The charge distribution and electronic structure of a material's molecules are fundamental to determining its physical and chemical properties.^[^ ^31^, ^32^ ^]^ To further investigate these characteristics, this section integrates results from ReaxFF molecular dynamics simulations with quantum chemical calculations based on density functional theory (DFT). Several representative solid‐phase organic molecules—namely C_5_H_8_O_3_, C_6_H_8_O, C_8_H_8_, and C_9_H_12_O_2_—were selected as typical products of epoxy resin cracking under electric field influence. Quantum chemical calculations were performed using Gaussian 16 B01, with geometric optimization and energy level computations conducted under the RB3LYP hybrid density functional and the 6‐31G(d) basis set.^[^ ^33^, ^34^ ^]^ To analyze the electronic structures, we employed the Multiwfn quantum chemical wave function analysis package and the VMD visualization tool.^[^ ^35^ ^]^ These platforms enabled visual examination of the energy level orbitals and electrostatic potential surfaces of both intact and decomposed epoxy resin molecules. Characteristic molecular structures before and after crosslinking and cracking were visualized using GaussView 6.0, as shown in Figure S21 (Supporting Information).

#### 2.6.1 Orbital Energy Levels of Epoxy Resin and Its Cracking Product Molecules

**Figure 8:**
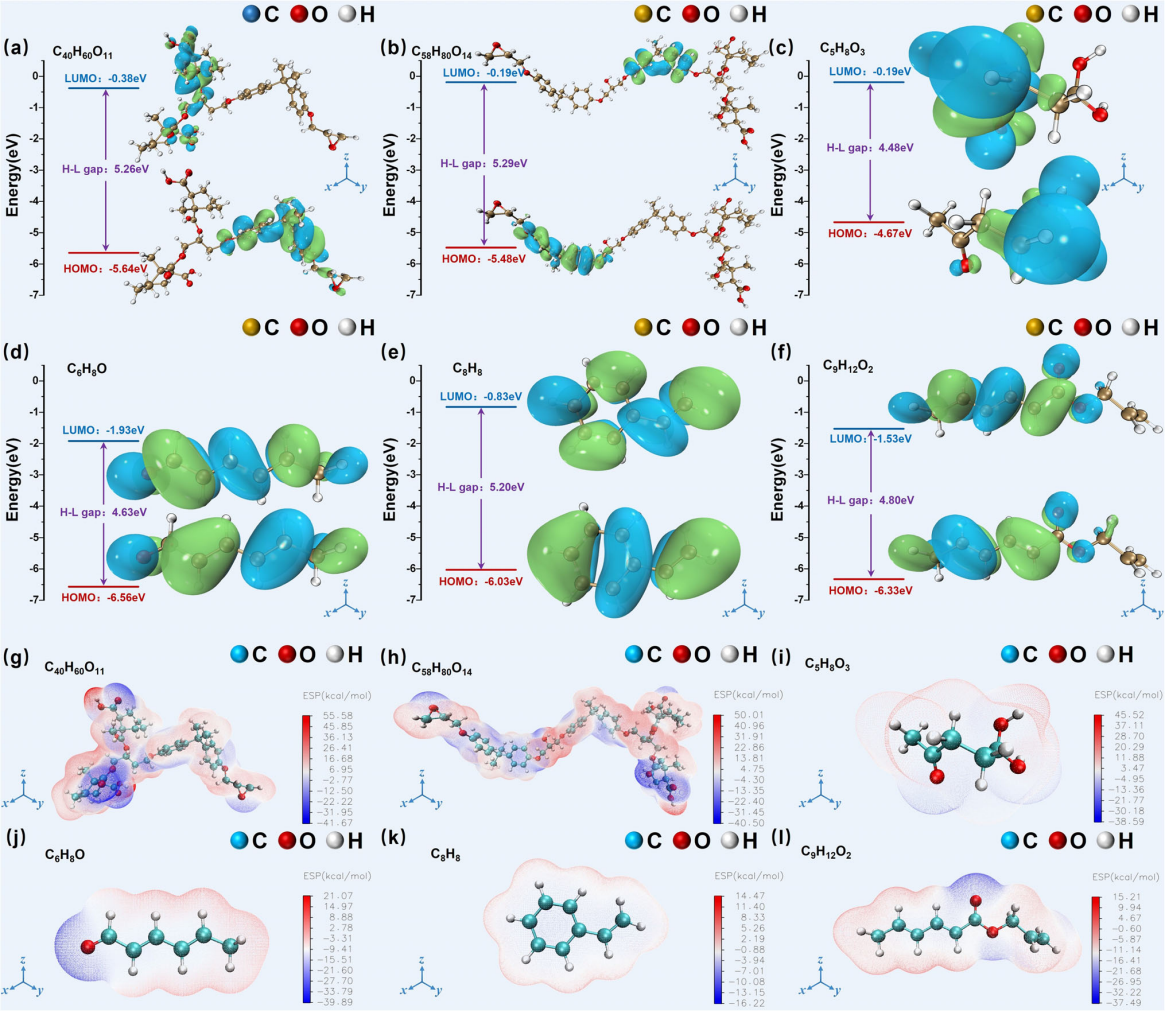
Orbital energy level distribution diagrams of epoxy resin molecules: a) when *n* = 0 in DGEBA and b) when *n* = 1 in DGEBA; orbital energy level distribution diagrams of thermally cracked products: c) C_5_H_8_O_3_, d) C_6_H_8_O, e) C_8_H_8_, and f) C_9_H_12_O_2_. Electrostatic potential energy distribution diagrams of epoxy resin molecules: g) when *n* = 0 in DGEBA and h) when *n* = 1 in DGEBA; electrostatic potential energy distribution diagrams of thermally cracked products: i) C_5_H_8_O_3_, j) C_6_H_8_O, k) C_8_H_8_, and l) C_9_H_12_O_2_.

As shown in **Figure** **8**a, the lowest unoccupied molecular orbital (LUMO) energy level of the epoxy resin (EP) molecule formed by the crosslinking of *n* = 0 DGEBA and MHHPA is −0.38 eV, while the highest occupied molecular orbital (HOMO) energy level is −5.64 eV, yielding a bandgap of 5.26 eV. In Figure 8b, the LUMO energy level of the EP molecule formed by the crosslinking of *n* = 1 DGEBA and MHHPA is −0.19 eV, and the HOMO energy level is −5.48 eV, corresponding to a bandgap of 5.29 eV. Similarly, the bandgap widths of small organic molecules such as C_5_H_8_O_3_, C_6_H_8_O, C_8_H_8_, and C_9_H_12_O_2_, shown in Figure 8c–f, are 4.48, 4.63, 5.20, and 4.80 eV, respectively. These results demonstrate that the bandgap widths of solid organic molecules generated via epoxy resin degradation are generally smaller than those of the intact EP molecules. Previous studies have shown that a smaller HOMO–LUMO energy gap implies a smaller energy barrier between occupied and unoccupied orbitals, leading to a lower excitation threshold for molecular transitions.^[^ ^36^, ^37^ ^]^ According to the Jablonski energy level diagram,^[^ ^38^ ^]^ the fluorescence generation process in materials can be described in detail, as illustrated in Figure S4 (Supporting Information). Typically, fluorescence emission follows the principles of the Stokes shift, where molecules absorb photons of shorter wavelengths and subsequently emit photons of longer wavelengths. As a result, the energy of the emitted fluorescence is lower than that of the absorbed excitation radiation. In the case of epoxy resin molecules, their relatively large HOMO–LUMO bandgap limits the possibility of electronic excitation from the ground state to the excited state. Consequently, absorbed energy is primarily dissipated through non‐radiative processes such as internal conversion or vibrational relaxation, and no fluorescence emission is observed under normal excitation conditions.

However, in regions affected by electrical treeing, the electrothermal degradation of the epoxy resin generates small organic molecules with significantly narrower HOMO–LUMO gaps. These smaller energy differences make radiative transitions more favorable.^[^ ^39^ ^]^ This structural transformation enhances the fluorescence quantum yield of the system, leading to intense fluorescence emission under laser excitation. Moreover, the disruption of the polymer's original crystallinity in the electrical tree region further facilitates radiative decay. As a result, these regions exhibit strong autofluorescence emission, attributable to the modified electronic and molecular structure caused by thermal and electrical stress.

#### 2.6.2 Electrostatic Potential Energy of Epoxy Resin and Its Cracking Product Molecules

To perform an in‐depth analysis of the electronegativity and charge distribution characteristics of epoxy resin molecules and the solid organic small molecules generated through electrothermal coupling‐induced cracking, we utilized the Multiwfn program in conjunction with VMD visualization tools. These were employed to examine the spatial variation in electrostatic potential energy across molecular structures. Figure 8g–l presents the electrostatic potential energy distribution maps for both epoxy resin molecules and their cracking products. In these visualizations, red represents regions of positive electrostatic potential energy, while blue indicates regions of negative potential energy. From Figure 8g,h, it is evident that in pure epoxy resin molecules, regions near carbon atoms generally possess positive potential energy, whereas those near oxygen atoms are characterized by negative potential energy. This charge asymmetry is especially pronounced at monomer junctions, where the potential energy is significantly lower than in the central segments of the molecule. Additionally, the MHHPA monomer region exhibits higher potential energy than the DGEBA monomer region, which may be attributed to differences in atomic electronegativity within the molecular framework.

Further analysis of Figure 8g–l shows that both the maximum and minimum absolute electrostatic potential values for epoxy resin molecules are substantially higher than those observed in the thermal degradation products, including C_5_H_8_O_3_, C_6_H_8_O, C_8_H_8_, and C_9_H_12_O_2_. This indicates that epoxy resin molecules possess a more pronounced electron cloud polarization and more uneven charge distribution. Such polarization may destabilize the excited state, increasing the likelihood of non‐radiative decay via internal conversion or intersystem crossing, which in turn suppresses fluorescence intensity. Moreover, the symmetry of the electrostatic potential energy distribution also significantly influences fluorescence intensity. Epoxy resin molecules display a relatively symmetric electrostatic potential energy distribution, which may enforce symmetry‐based selection rules that restrict radiative electronic transitions, resulting in weaker fluorescence. In contrast, cracking product molecules such as C_5_H_8_O_3_, C_6_H_8_O, C_8_H_8_, and C_9_H_12_O_2_ exhibit asymmetric electrostatic potential distributions. This asymmetry may break the symmetry constraints, enhance the probability of allowed electronic transitions, and thereby increase autofluorescence intensity.

In summary, based on the characterization results presented in Section 2.4, molecular dynamics simulations in Section 2.5, and quantum chemical simulations in Section 2.6, the mechanism underlying autofluorescence emission from defect structures such as electrical trees in polymer materials—using epoxy resin as a model—can be elucidated. Under the influence of an electric field of sufficient intensity, molecular structural rearrangements occur in the defective regions of the polymer matrix, leading to the formation of numerous organic small molecules containing functional groups such as C═C and C═O. Macroscopic tests performed on these defective areas confirm that at least three types of fluorescent molecules contribute to the observed autofluorescence phenomenon. These regions exhibit pronounced circular dichroism in the visible spectrum range of 320–800 nm and display a peak redshift in the UV–vis absorption spectrum within the 275–335 nm range. At the microscopic level, the autofluorescence effect originates from electronic transitions such as *n*–π* and π–π* between chemical bonds (e.g., C═O, C═C) within aggregated molecular systems in the defective regions. These π‐electron‐rich species tend to cluster under the coupled effects of electrical and thermal stimulation. When the distance between constituent atoms falls below the sum of their van der Waals radii, effective electronic coupling occurs, resulting in π‐electron delocalization. Furthermore, several of the small organic molecules produced—such as C_5_H_8_O_3_, C_6_H_8_O, C_8_H_8_, and C_9_H_12_O_2_—exhibit relatively narrow HOMO–LUMO bandgaps, making them more susceptible to radiative transitions. Additionally, these molecules possess asymmetric electrostatic potential energy distributions, which can disrupt symmetry selection rules, thereby increasing the likelihood of electronic transitions and enhancing fluorescence intensity. The combination of effective electronic interactions and extended delocalization due to molecular aggregation facilitates increased energy release through radiative transitions when the material is exposed to laser excitation. This synergistic mechanism gives rise to the distinctive autofluorescence emission observed in polymer defect regions, particularly in the presence of electrical trees.

### 2.7 Phase Field Simulation of Electrical Trees

**Figure 9:**
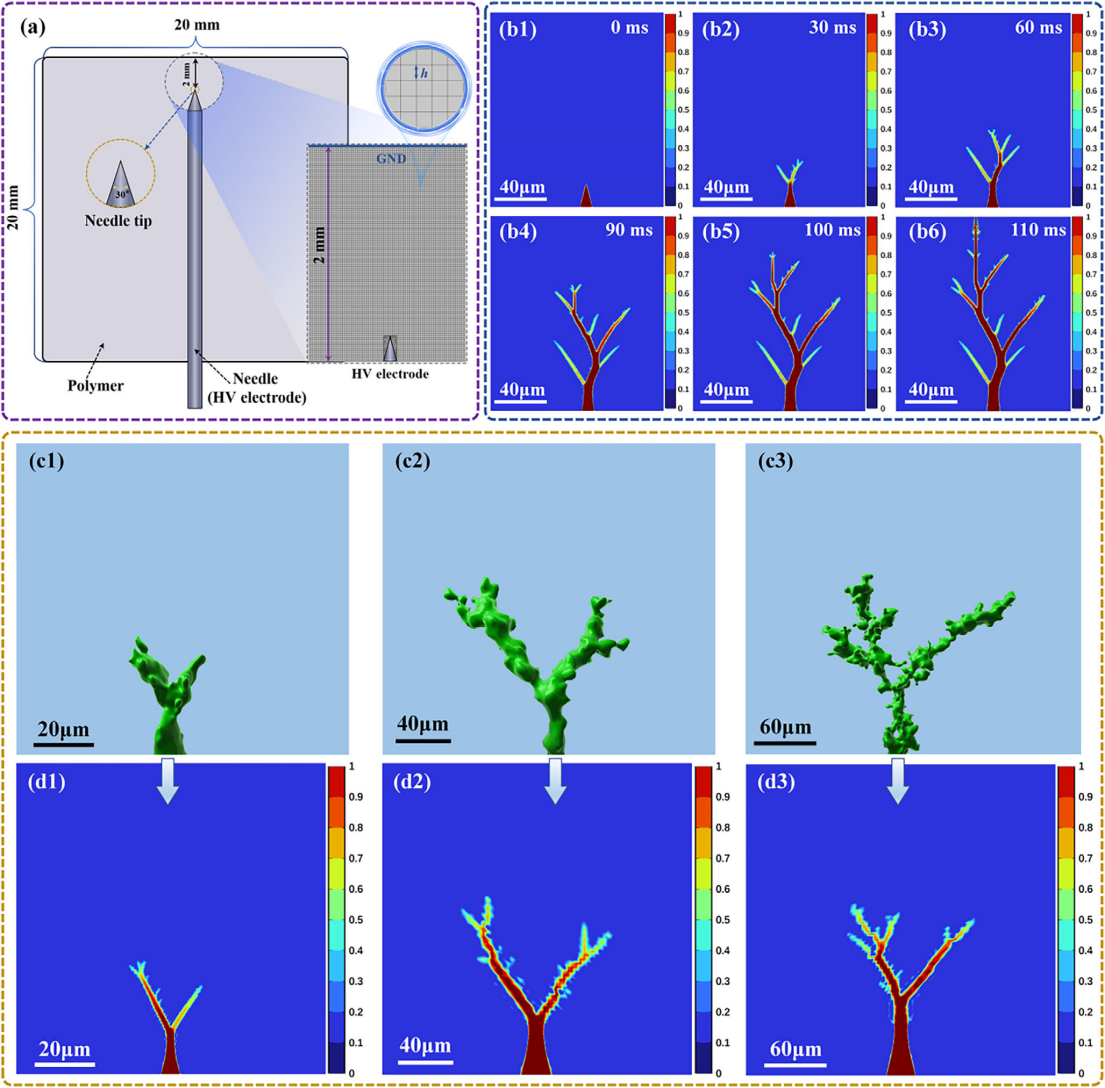
a) Geometric parameters of the phase field model for electrical tree development; b1–b6) Simulated time‐evolution images; c1–c3) Autofluorescence‐based 3D reconstructions of electrical tree defects; d1–d3) Corresponding simulation results based on experimental geometries.

Based on the geometric data obtained from three‐dimensional autofluorescence reconstruction of electrical trees, a phase field simulation model was developed to study the evolution of electrical tree defects under an applied electric field. In this study, an epoxy resin sheet sample with dimensions of 20 mm × 20 mm × 1 mm was used, with a pre‐inserted needle electrode. A high voltage (HV) of 12 kV was applied to the needle electrode using the power supply setup described in Figure S1 (Supporting Information), thereby inducing electrical trees within the epoxy resin. The needle tip was positioned 2 mm from the ground (GND) electrode, and its cross‐section had a sharp angle of 30°. According to the actual geometric model parameters, the size, simulation parameters, and mesh division of the phase field model were configured as shown in **Figure** **9**a. The phase field model simulation enables the spatiotemporal analysis of electrical tree development by introducing a phase field variable *η*(*p*, *t*), which is a function of spatial position *p* and time *t*. The evolution of *η* reflects the extent of material degradation: regions where *η*(*p*, *t*) = 0 represent undamaged, intact material; regions where *η*(*p*, *t*) = 1 represent fully damaged zones; and regions where 0 < *η*(*p*, *t*) < 1 indicate partially damaged areas, which are considered part of the electrical tree when compared to intact zones. These interpretations are based on the fundamental properties of the phase field model,^[^ ^40^, ^41^ ^]^ and the simulation results are displayed in Figure 9b1–b6,d1–d3. In Figure 9b1–b6, the full development process of electrical tree defects—from initial inception to eventual breakdown—is simulated over a time period of 110 ms. The simulation reveals that defect propagation is relatively slow during the initial 60 ms. However, between 90 and 110 ms, the electrical tree rapidly expands, culminating in material breakdown. This pattern of accelerated progression is highly consistent with the experimentally observed growth behavior of electrical trees. Further details on the temporal evolution of the phase field and electric field variables during the simulation are provided in Figure S22 and Movie S4 (Supporting Information). To verify the accuracy of the simulation, the actual three‐dimensional dimensions of the electrical trees were obtained via autofluorescence‐based 3D imaging. These data were reconstructed and imported into the phase field model, as shown in Figure 9c1–c3. A comparison between Figure 9c1–c3 and the phase field simulation outputs in Figure 9d1–d3 reveals a close match in the size and morphology of the electrical trees. This correspondence indicates that the phase field model effectively captures the dynamic evolution characteristics of electrical tree defects. Moreover, this finding suggests that early‐stage electrical tree parameters can be introduced into the phase field model to simulate and potentially predict the real development trajectory of such defects. It should be noted, however, that the simulated growth rate of electrical trees in this study is faster than the actual propagation speed observed experimentally. While the phase field simulation provides reliable predictions regarding the general size and structural pattern of electrical trees, slight discrepancies may exist between the modeled and real‐world three‐dimensional morphologies and breakdown timelines.

To further elucidate the principles of phase‐field simulations, the electrical tree propagation region, based on dielectric degradation theory, can be modeled as a region with a high dielectric constant. The development of an electrical tree is treated as a phase transition process of the phase‐field variable associated with the dielectric constant under the influence of an external electric field. This phase transition is energy‐driven, and the total energy equation describing the microscopic system is presented in Equation (2), which represents the total free energy in the phase‐field model.^[^ ^40^ ^]^

(2) $$F=\int_{V}\left[f_{\mathrm{sep}}\left(\eta \left(p,t\right)\right)+\frac{1}{2}c\gamma {\left|\nabla \eta (p,t)\right|}^{2}+f_{\mathrm{elec}}\left(p,t\right)\right]dV$$

In Equation (2), *f*_sep_ denotes the free energy density, the second term represents the gradient energy density, *γ* is the gradient energy coefficient, *c* is the diffusion coefficient, and *f*_elec_ represents the electric field energy density. All three terms in Equation (2) are functions of the phase‐field variable.

The free energy density is typically expressed using a one‐dimensional finite‐depth potential well, as shown in Equation (3).

(3) $$f_{\mathrm{sep}}\left(p,t\right)=\alpha \eta {\left(p,t\right)}^{2}{\left[1-\eta \left(p,t\right)\right]}^{2}$$

In Equation (3), *α* denotes the energy barrier coefficient.

The electric field energy density is expressed by Equation (4).

(4) $$f_{\mathrm{elec}}\left(p\right)=-\frac{1}{2}\varepsilon_{0}\varepsilon_{r}\left(p\right)E\left(p,t\right)\cdot E\left(p,t\right)$$

In Equation (4), *ε*_0_ is the vacuum permittivity, *ε_r_* is the relative permittivity of the dielectric medium, and *E*(*p*,*t*) is the electric field strength at location *p* and time *t* within the dielectric region.

Based on the fundamental equations of electrostatics, the electric field strength at each point in the dielectric can be determined by solving the Poisson equation within the computational domain. The electrostatic Poisson equation is given in Equation (5).

(5) $$\nabla \cdot [\varepsilon_{r}\left(p,t\right)\nabla \varphi \left(p,t\right)]=0$$

In Equation (5), the electric potential variable *φ*(*p*,*t*) and the phase‐field variable *η*(*p*,*t*) together form the dependent variables in the phase‐field simulation of electrical tree development. These variables are linked via the relative permittivity of the dielectric medium. In electrostatic fields, variations in relative permittivity influence the distribution of electric field strength, thereby affecting the total free energy in the phase‐field model.

Channels formed during dielectric degradation are typically semi‐conductive or conductive, with their relative permittivity gradually transitioning from the initial insulating state *ε*_I_ to the fully degraded terminal state *ε*_D_ as electric field application time increases. The evolving state of the channel during degradation is represented by a transfer function *fε* in the phase‐field model, as shown in Equation (6).

(6) $$\varepsilon_{r}\left(r\right)=f_{\varepsilon }\left[\eta \left(p,t\right)\right]\varepsilon_{D}+\left\{1-f_{\varepsilon }\left[\eta \left(p,t\right)\right]\right\}\varepsilon_{I}$$

In Equation (6), *f*_ε_ describes the variation of the dielectric constant of the medium with respect to the phase‐field variable. It is a material‐specific characteristic that depends solely on the physical state of the medium.

## 3 Conclusion

In this work, we investigated autofluorescence generated from internal defects in polymers under controlled electric fields. This phenomenon was reproducible across multiple polymer matrices and various defect types, including electrical trees, bubbles, and metal particles. Building on this, we established an in situ characterization method that enables high‐resolution, in situ, three‐dimensional imaging of internal microstructures in polymeric systems without the need for exogenous fluorescent labels. The mechanism underlying the spontaneous fluorescence phenomenon is hypothesized as follows. Preliminary evidence from fluorescence lifetime microscopy and multispectral analyses suggests that electric field exposure induces molecular dissociation in polymers, resulting in the formation of autofluorescent oligomers. Multiscale simulations combining ReaxFF and DFT calculations revealed that electric field‐induced polymer chain cleavage generates π‐conjugated oligomers with reduced HOMO–LUMO energy gaps and flattened electrostatic potential profiles. These molecular changes confer enhanced conformational rigidity and facilitate extended π‐orbital delocalization, thereby favoring radiative decay pathways and increasing fluorescence quantum yield. Critically, this approach enables high‐resolution, three‐dimensional, and in situ mapping, serving as a valuable complement to conventional methods such as SEM and XCT. As such, it opens new avenues for non‐destructive investigations into the formation of defects (e.g., electrical trees) and early‐stage degradation phenomena in polymer systems.

## 4 Experimental Section

### Preparation of Epoxy Resin Materials

Epoxy resin samples were prepared as follows: Component A (bisphenol A epoxy resin, E51) and component B (methyl hexahydrophthalic anhydride, MHHPA) were each placed into separate beakers and heated in a 60 °C water bath with continuous stirring for 20 min to ensure thorough mixing. The components were combined at a ratio of 1:0.85 (A:B) and maintained at 60 °C. The mixed solution was then transferred to a constant‐temperature vacuum drying oven and degassed at a pressure of −0.10 MPa for 30 min.

Meanwhile, a metal mold was preheated to 150 °C and sprayed with a release agent to facilitate demolding. After degassing, the mixture was poured into the mold, cured at 100 °C for 2 h, and subsequently at 120 °C for 3 h. The final product was an epoxy resin sheet sample with a thickness of 1 mm.

### Preparation of XLPE, RTV, and PDMS Materials

Polyethylene particles from high‐voltage cable insulation (peroxide‐crosslinked XLPE) were preheated in a plate vulcanizing machine at 120 °C for 10 min. Crosslinking was then carried out at 180 °C under 20 MPa for 20 min. A needle electrode with a curvature radius of 25 µm was embedded into the material before crosslinking. After cooling to room temperature, a flat, uniform XLPE sample was obtained.

Components A and B of liquid RTV were mixed at a 1:1 ratio and stirred mechanically at 200 rpm for 20 min. The mixture was then degassed multiple times at −0.10 MPa in a vacuum drying oven at 20 °C. Simultaneously, a metal mold was heated to 80 °C and coated with a release agent. After degassing, the mixture was poured into the mold and cured at 80 °C for 3 h, resulting in a semi‐transparent RTV sheet sample with a thickness of 1 mm.

Component A (PDMS base) and component B (curing agent) were mixed at a 10:1 ratio and stirred mechanically at 200 rpm for 25 min. The mixture was then degassed at −0.10 MPa multiple times. A mold was heated to 80 °C and treated with a release agent. After degassing, the PDMS mixture was poured into the mold and cured at 80 °C for 4 h to produce a PDMS sheet sample with a thickness of 1 mm.

For all materials, the depth of needle electrode implantation and the distance between the needle tip and ground electrode were precisely controlled to construct a high‐voltage needle–plate electrode configuration, ensuring consistent initiation of electrical tree formation under power‐frequency voltage.

### Simulation Method

Molecular dynamics simulations were conducted using Materials Studio, LAMMPS, and the ReaxFF reactive force field, employing the established C/H/O interaction parameters. Temperature control was achieved using a thermostat with a relaxation time of 20 fs. The equations of motion were integrated via the velocity‐Verlet algorithm. Throughout the ReaxFF molecular dynamics simulations, atomic coordinates were recorded for post‐simulation analysis.

Quantum chemical calculations for epoxy resin molecules were performed using Gaussian 16 B01. Initially, Gaussian molecular structures were constructed for the two primary molecular components of the epoxy resin system. Using the hybrid density functional RB3LYP and the 6‐31G(d) basis set, geometry optimizations and energy calculations were carried out for the crosslinked epoxy resin molecules, both prior to and following electric field application. Visualization of molecular orbitals and conformational structures before and after field exposure was achieved using the Multiwfn (Multifunctional Wavefunction Analyzer) program in combination with VMD (Visual Molecular Dynamics) software.

Phase field simulations were performed using COMSOL Multiphysics 6.3. The temporal and spatial evolution of the phase field variable *η*(*p*, *t*) was governed by the Allen–Cahn equation. The characteristic length scale of the simulation domain was defined as $d_{0}=\sqrt{\gamma /\alpha }$; to balance computational accuracy and efficiency, the maximum grid size was set to 10 *d*_0_. Based on the characteristic time scale definition *t*_0_ *=* 1/(*L*_0_ *α*), the maximum solver time step was set to 0.05 *t*_0_. To simulate the stochastic evolution of electrical tree growth in solid dielectric media, a random coefficient of 0.3 was introduced into the free energy density term in the phase field evolution equation.

## Conflict of Interest

The authors declare no conflict of interest.

## Supporting information



Supporting Information

Supplemental Movie 1

Supplemental Movie 2

Supplemental Movie 3

Supplemental Movie 4

## Data Availability

The data that support the findings of this study are available from the corresponding author upon reasonable request.
