# Supplementary material for: Electric Field‐Stimulated Autofluorescence for In Situ 3D Characterization of Polymer Defects
Source: Adv Sci (Weinh). 2025 Sep 19;12(45):e11290. doi: 10.1002/advs.202511290 (PMC12677673; doi:10.1002/advs.202511290)
Supplement: Supplementary file 1 — Supporting Information [file ADVS-12-e11290-s001.docx]

**Supporting Information**

**Title: Electric Field-Stimulated Autofluorescence for In Situ 3D Characterization of Polymer Defects**

*Chaolu Niu,^1^ Potao Sun,^1^* Wenxia Sima,^1^ Tao Yuan,^1^ Ming Yang,^1^ Yuhang Yang,^1^ Yuxiang Mai,^1^ Qin Deng,^2^ and Yu Zhang^3^*

*^1^*State Key Laboratory of Power Transmission Equipment Technology, Chongqing University, Chongqing 400044, People’s Republic of China.

*^2^*School of Physics, Chongqing University, Chongqing 400030, People’s Republic of China.

*^3^*Analytical and Testing Center, Chongqing University, Chongqing 400030, People’s Republic of China.

*Corresponding E-mail: sunpotao@cqu.edu.cn

1. **High-Voltage Power Frequency Test Platform**

The high-voltage power frequency test platform comprises a control console, an oscilloscope, a power frequency experimental transformer, a voltage regulator, protection resistors, a voltage divider, a real-time observation optical microscope, grounding devices, and experimental samples. The electrode parts of the test unit in this platform can be classified into two types: needle-plate electrodes and plate-plate electrodes, as shown in Figures S1 and S2, respectively. Firstly, this work prepared sheet samples of different materials such as EP, XLPE, RTV, and PDMS, each with dimensions of 20 mm × 20 mm × 1 mm. Taking the epoxy resin material as an example, needle electrodes, bubbles, and metal particles were pre-placed inside the epoxy resin material to generate electrical trees and simulate different types of defects. For the XLPE, RTV, and PDMS sheet samples, needle electrodes were pre-placed inside to generate electrical tree defects.

When pre-placing needle electrodes inside the aforementioned materials, the tip of the needle electrode inside the material is 2 mm away from the ground electrode, and the cross-section of the needle tip presents a sharp angle of 30°. The selected high-voltage power frequency test platform is shown in Figure S1. When pre-placing bubbles and metal particles inside the above-mentioned materials, the radius of the column electrode in the test unit of this platform is 25 mm, with a chamfer of 3 mm radius at the edge, and the sample is a 1 mm thick slice of material. The selected high-voltage power frequency test platform is shown in Figure S2. To prevent flashover along the surface of the sample during the pressure application process, which may affect the experimental results, this paper immerses both the sample and the electrode in clean 18# mineral insulating oil. In the experiments of this paper, the step-up voltage method is adopted, and an AC voltage is applied to the sample at a rate of 0.5 kV/s until a significant autofluorescence effect appears in the defect area of the sample.


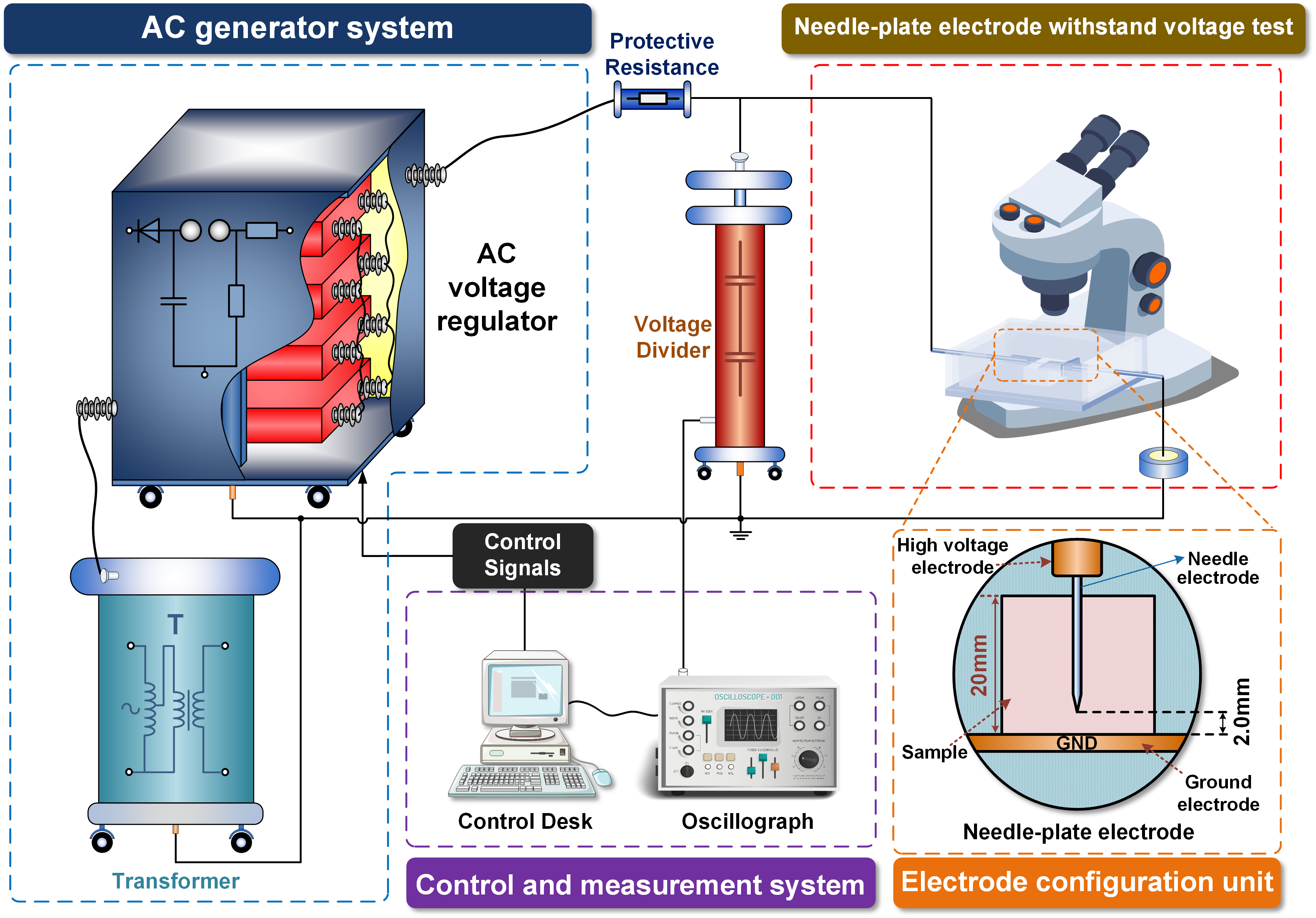


**Figure S1.** Schematic diagram of the power frequency transformer experimental platform.


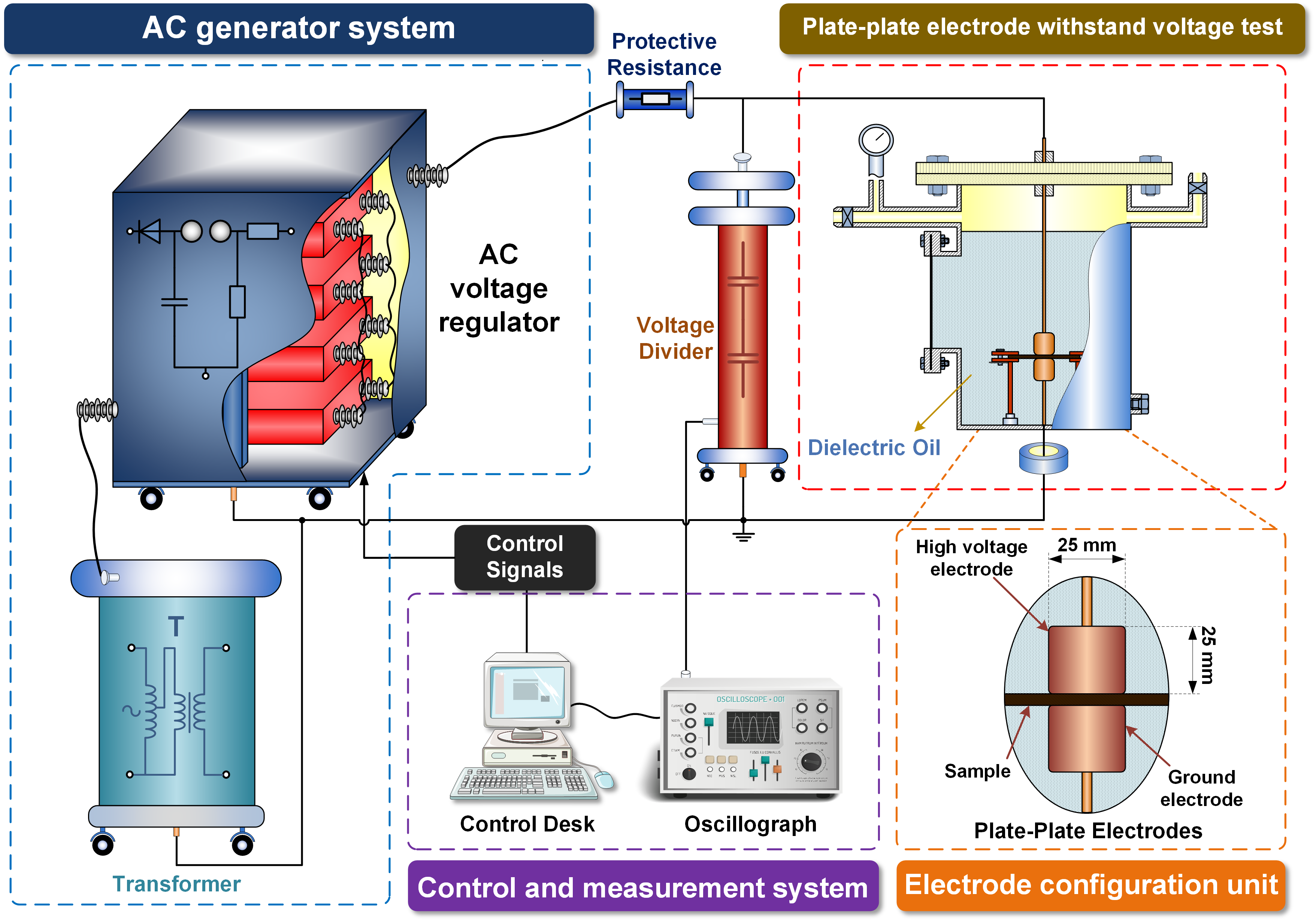


**Figure S2.** Schematic diagram of the power frequency transformer experimental platform.

1. **Principle of Fluorescence-Excited Emission**

Fluorescence excited by the characteristic structure of materials is an in-situ fluorescence, which is expected to solve the problem of nondestructive three-dimensional characterization. When a substance is excited by different forms of energy (light, electricity, bioenergy, physical energy, etc.), it emits colorful fluorescence. Current research at home and abroad has concluded that regardless of the type of energy causing the excitation, the mechanism of light emission is the same. As shown in the luminescence mechanism diagram in Figure S3, during the process of absorbing incident light energy for excitation, the photon energy is transferred to the molecules of the substance, the molecules are excited, and electrons transition from the ground state to the excited state, forming electronically excited molecules. However, the excited state of electrons is unstable. When electrons return to the ground state, they lose energy through radiative and non-radiative transitions, among which the fastest pathway with the shortest excited state lifetime dominates.


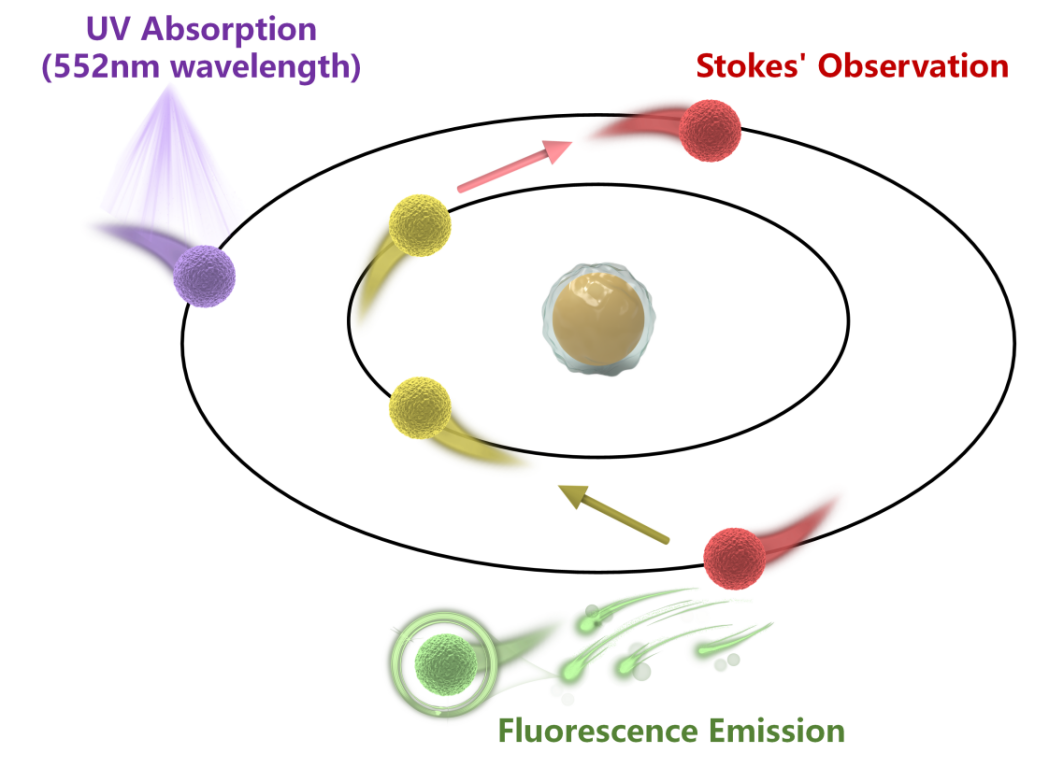


**Figure S3.** Diagram of the fluorescence emission mechanism.

As shown in Figure S4, the Jablonski diagram describes the dynamic process of fluorescence from excitation to emission^[38]^. Generally speaking, fluorescence emission conforms to the Stokes shift, meaning that molecules absorb photons of shorter wavelengths and then emit photons of longer wavelengths; the emission wavelength is always greater than the excitation wavelength. The energy absorbed by solids (absorption) exceeds that of the radiated photons (emission), so the emission spectrum is shifted towards lower energy (redshift) compared to the absorption spectrum. As shown in the Jablonski diagram in Figure S4, the dynamic process of fluorescence from excitation to emission mainly includes the following aspects: (1) From the ground state S0 to the excited state. Due to the action of external excitation light, electrons are forced to transition from the ground state S0 to an excited state above the lowest vibrational level S1 of the first excited singlet state. (2) From the lowest vibrational level S1 of the first excited singlet state to the ground state S0. Excited-state electrons rapidly reach the lowest vibrational level of the first electronically excited singlet state S1 through internal conversion and vibrational relaxation processes. At this time, the electrons are still in an unstable state, and when returning to the ground state S0, energy is released in the form of radiative transitions, mainly emitting fluorescence. (3) From the lowest vibrational level S1 of the first excited singlet state to the first electronically excited triplet state T1. Electrons at energy level S1 transition to the triplet state T1 through intersystem crossing and vibrational relaxation. (4) From the first electronically excited triplet state T1 to the ground state S0: Electrons in the triplet state T1 release part of their energy in the form of radiative transitions, emitting minor phosphorescence. In Figure S4, electronic states are represented by horizontal lines, with thick lines indicating the corresponding vibrational ground states. Transitions within and between electronic states occurring at different times can be non-radiative (indicated by red arrows) or radiative (indicated by green and yellow arrows). Fluorescence occurs on the nanosecond scale. In most cases, the excitation and emission wavelengths produce a Stokes shift.


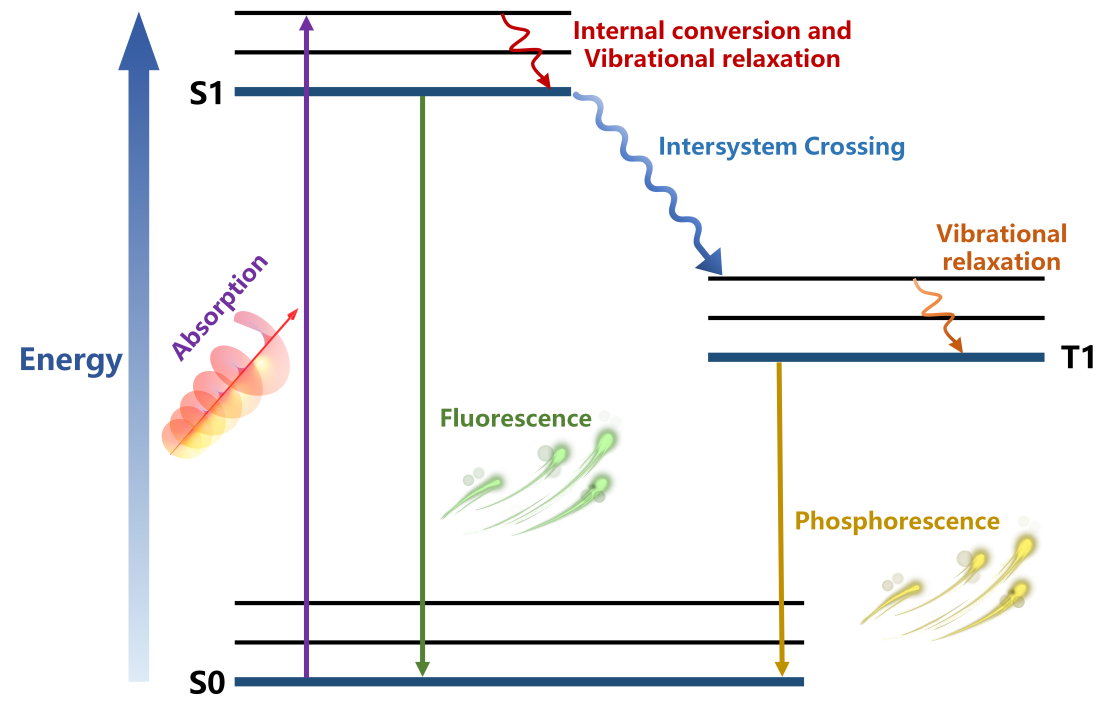


**Figure S4.** Jablonski molecular energy level diagram. S0: Ground state; S1: First excited singlet state; T1: First excited triplet state.

**References：**

[38] T. J. Penfold, E. Gindensperger, C. Daniel, C. M. Marian, *Chem. Rev.* **2018**, *118*, 6975-7025.

1. **Detailed Description of the Confocal Fluorescence Imaging Platform**

As illustrated in Figure S5, confocal fluorescence imaging was employed. A point light source illuminated the sample, generating a well-defined microscopic spot on the focal plane. Fluorescent emissions generated upon excitation were collected by the objective lens and retroreflected along the incident optical path toward a polarization beam splitter composed of dichroic mirrors. The resulting fluorescence signal was subsequently directed to the detector. Both the illumination and detection pathways incorporated pinholes (illumination pinhole and detection pinhole), each with a geometric aperture of approximately 100–200 nm. These pinholes were conjugate relative to the focal plane, meaning that the focused spot could be precisely mapped through an optical train onto both the illumination and detection pinholes. This configuration ensured that only photons originating from the focal plane were spatially confined within the detection pinhole, while out-of-focus scattered light from regions above or below the focal plane was effectively rejected.

Following the application of a 12 kV voltage for 18 minutes, a 552 nm laser was selected to irradiate the electrical tree region. The sample underwent point-by-point scanning with the laser beam. During this process, fluorophore molecules exhibiting fluorescent responses underwent transitions from the ground state to the excited state. Subsequently, these excited molecules returned to the ground state via spontaneous emission, releasing fluorescent photons. A photomultiplier tube positioned behind the detection pinhole captured these photons sequentially, converting them into digital signals transmitted to a computer system. Through raster scanning, confocal image reconstruction corresponding to the scanned spots was achieved, enabling the acquisition of two-dimensional (2D) confocal images across the entire xy focal plane.

The sample was progressively scanned layer-by-layer along the z-axis (perpendicular to the sample surface, as depicted in Figure S5), yielding 2D morphological data at varying depths. These cross-sectional images were computationally processed to reconstruct a three-dimensional (3D) representation of the electrical tree structure. Additionally, brightfield images of the specimen were captured using a charge-coupled device (CCD) camera to assist in spatially correlating fluorescence signals with interface features. Leveraging the principle of stimulated emission-induced spontaneous fluorescence, this approach enabled high-resolution characterization of the 3D architecture of the electrical tree.

The confocal laser scanning microscopy (Leica Microsystems GmbH) utilized in this study demonstrated superior spatial resolution, achieving 140 nm precision in the xy-plane and 400 nm precision along the z-axis.


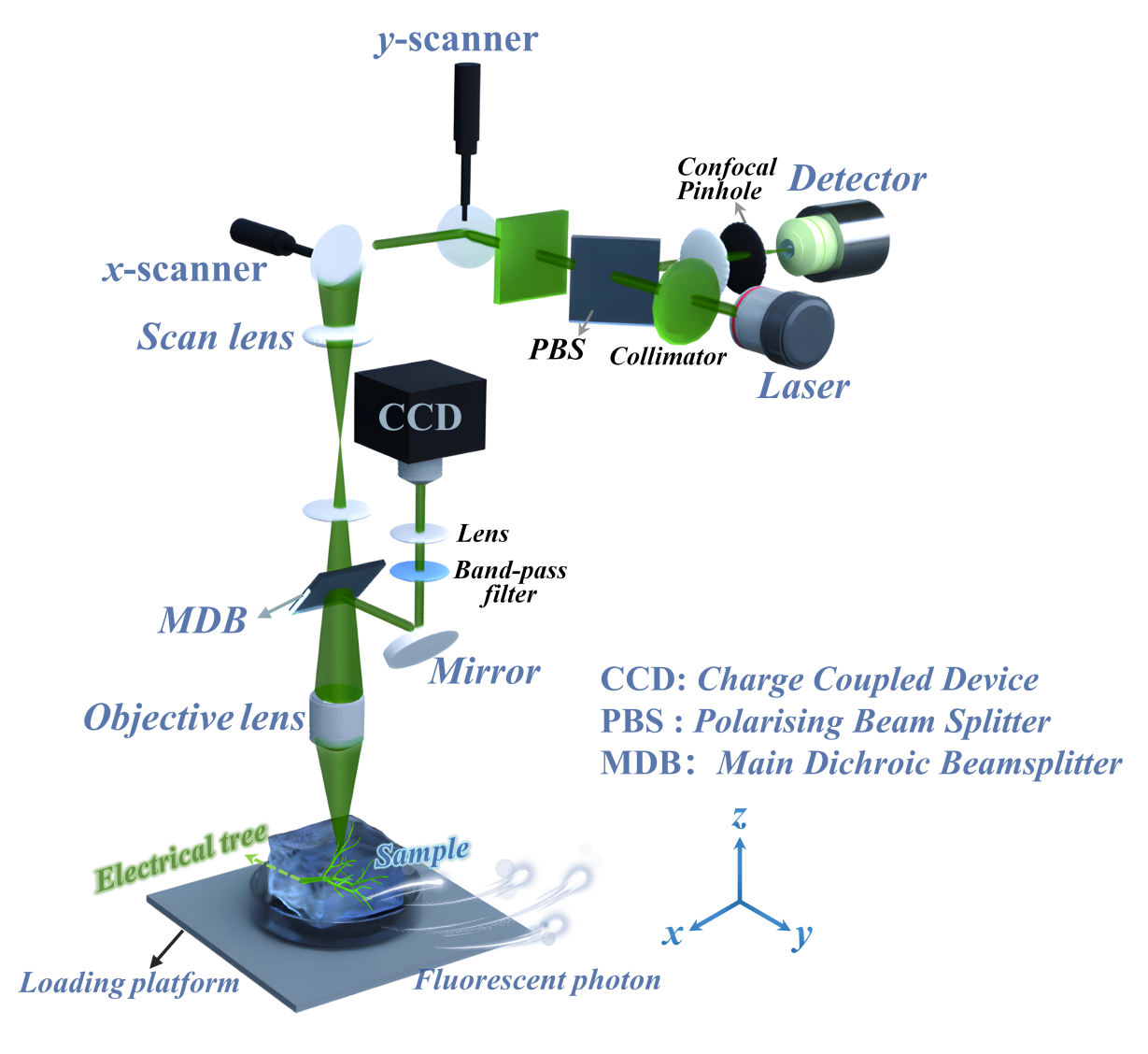


**Figure S5.** Structural schematic diagram of the confocal fluorescence imaging platform.

1. **Electric Field Distribution in Different Defects**

To analyze the universality of the autofluorescence effect on different types of defects inside materials, this paper selects epoxy resin (Epoxy resin, EP) sheet samples with dimensions of 20 mm × 20 mm × 1 mm as objects and presets needle electrodes, bubbles, and metal particles inside them to generate electrical trees and simulate different types of defects. After applying 12 kV voltage to the needle electrode in the epoxy resin sample using the high-voltage power supply shown in supporting information Fig. S1 and lasting for 18 minutes, an electrical tree was generated at the tip of the needle electrode inside the epoxy resin sample. At this time, the result of autofluorescence imaging of the excited radiation of the electrical tree is shown in Figure 3(a2) of the main text. However, after applying 12 kV voltage to the epoxy resin sample containing bubbles and metal particle defects using the high-voltage power supply shown in supporting information Fig.S2 and lasting for 18 minutes, no autofluorescence signal was detected. The applied voltage was further increased until voltages of 20 kV and 28 kV were applied to samples containing bubbles and metal particle defects (also lasting for 18 minutes), respectively). Autofluorescence signals were observed, and the results of autofluorescence imaging of the samples are shown in Figure 3(a2) and 3(b2) of the main text, respectively. We infer that due to the different geometric shapes of needle electrodes and bubbles and metal particle defects, there are differences in the electric fields around the defects when the same voltage is applied. Therefore, a simulation model was established to analyze the electric field distribution around needle electrodes, bubbles, and metal particles, as shown in supporting information Fig. S6. It can be seen that when voltages of 12 kV, 20 kV, and 28 kV are applied to them, the maximum field strengths near the defects are 171.26 kV/mm, 143.17 kV/mm, and 144.31 kV/mm, respectively. Moreover, the average field strength near the defects exceeds 100 kV/m, indicating that the threshold field strength for the phenomenon of field-induced self-excited fluorescence is the same in all three samples, which is 100 kV/mm.

**
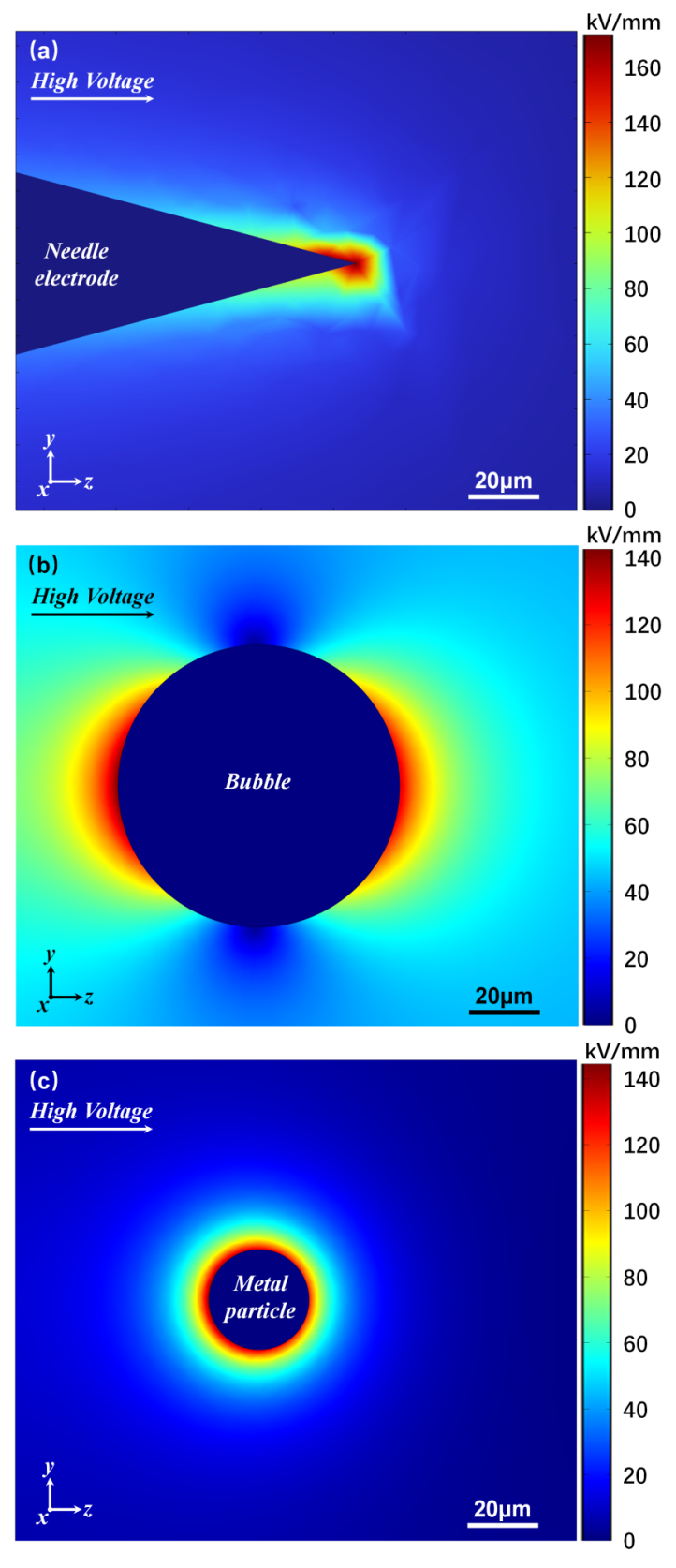
**

**Figure S6.** Electric field distribution near different types of defects inside polymers.

1. **Comparison of Optical Microscopy and Autofluorescence Imaging Effects**

In this work, the electrical trees inside epoxy resin were observed under both optical microscopy and confocal fluorescence microscopy, with the imaging effects shown in Figure S7. Under an optical microscope, it is almost impossible to observe micron-sized electrical tree channels due to low imaging accuracy, as shown in Figure S7(a). However, in the depth images of confocal autofluorescence imaging, we can clearly observe micron-sized electrical tree channels, with the detailed morphology of the imaged electrical trees shown in Figure S7(b).


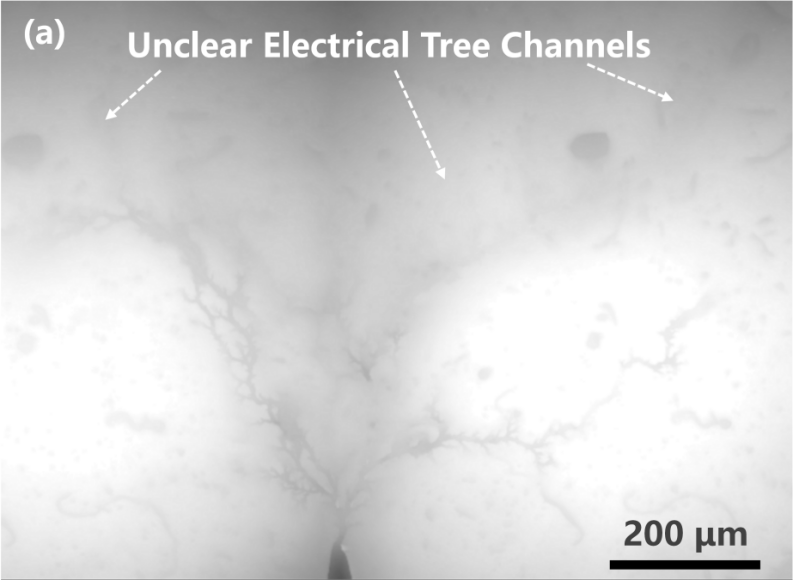


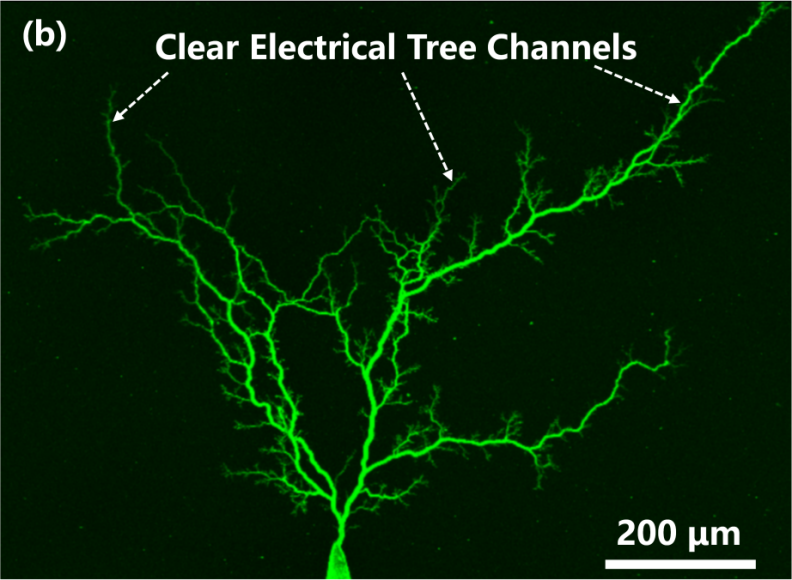


**Figure S7.** (a) Optical microscope depth of field map; (b) Fluorescence microscope depth of field map.

1. **Gaussian Filtering Process for Autofluorescence Background Signals**

During the three-dimensional imaging process of autofluorescence of defects such as electrical trees inside polymer materials, the confocal microscopy imaging system is affected by environmental interference or the background signal of the sample itself, resulting in excessive noise in the imaged electrical tree fluorescence morphology. This paper adopts a Gaussian filtering algorithm to remove background signals and avoid the interference of fluorescent noise on the autofluorescence imaging of electrical trees. The morphology of the electrical trees before and after Gaussian filtering is shown in Figure S8.


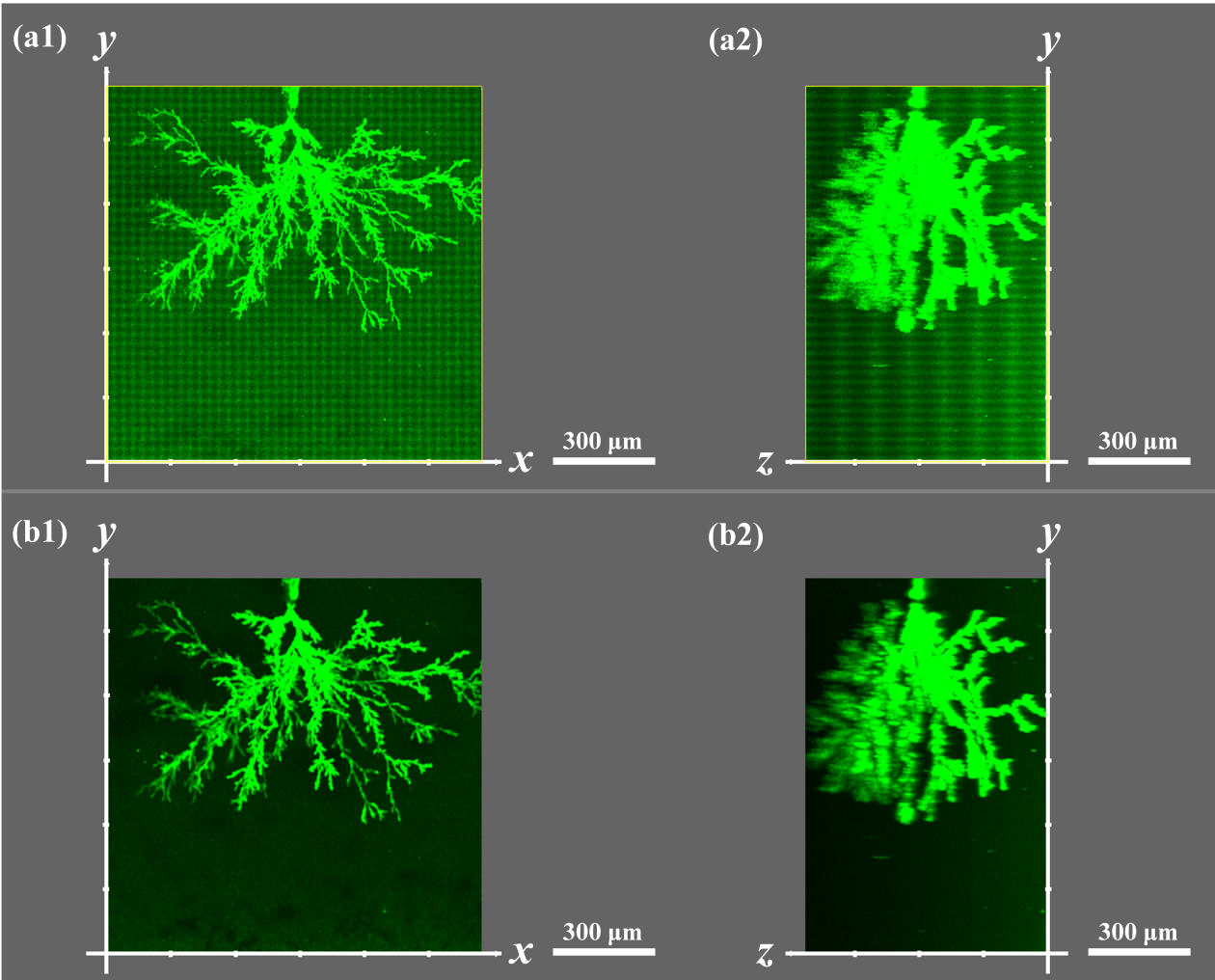


**Figure S8** Images of the results before (a1-a2) and after (b1-b2) Gaussian Filtering of the autofluorescence information in electrical tree.

1. **Three-Dimensional Reconstruction Process of Autofluorescence**

During the three-dimensional fluorescence imaging process of electrical trees inside epoxy resin, the morphology of electrical tree fluorescence presents varying degrees of loss due to environmental interference or background signals inherent to the sample itself. To address the issues of discontinuity or excessive noise in the imaging morphology of electrical tree fluorescence, it is necessary to perform spatial reconstruction of the electrical tree fluorescence morphology. Given that the morphology of electrical trees resembles dendritic structures and that the radius of electrical tree channels decreases with increasing growth length, exhibiting characteristics of larger starting radii and smaller ending radii, coupled with a clear intensity contrast between fluorescence and background signals, this paper utilizes an automatic path planning algorithm for three-dimensional reconstruction of electrical tree fluorescence morphology. The three-dimensional reconstruction process is shown in Fig. S9.

During reconstruction, the fluorescence signal path, starting and ending points of the electrical tree channel in the target area are first defined. Based on the characteristics of the electrical tree channel morphology, the maximum radius is obtained through statistical analysis of the fluorescence signal radius and defined as the starting point of the electrical tree, with the corresponding minimum radius defined as the endpoint. The maximum and minimum radii are used as the upper and lower threshold limits for the electrical tree path points, as shown in Figure S9(b). When obtaining the radius of the electrical tree, the "cross-sectional area approximation circle planning method" is employed. When the main axis (central axis) of the electrical tree is not well centered (such as in large irregular dendritic structures), the area of the green circle is equal to the cross-sectional area determined by the threshold, as shown in Figure S9(c). At this time, the radius of the green circle is defined as the channel radius of the fluorescent electrical tree. This method is applicable in all x, y, and z directions, achieving the most approximate three-dimensional reconstruction. After determining the connection path of the electrical tree, it is necessary to draw the contour of the electrical tree channel. Contour drawing is performed by boundary localization of fluorescent signals resembling spheres or ellipsoids along the electrical tree path. Based on the aforementioned premises, fluorescent signals within the target area can be divided into three categories: those above the maximum radius, below the minimum radius, and between these two values. Fluorescent signals with larger radii are defined as the starting points of the tree, those with smaller radii as the endpoints, and signals between the maximum and minimum values as connection points of the electrical tree path, as shown in Figure S9(d). Points between the low and high thresholds are defined as electrical tree channel path points. Finally, based on the starting points, endpoints, and path points, the electrical tree space scanning path is automatically obtained. During reconstruction, it is also necessary to avoid incorrect path connections. At this stage, it is necessary to set a maximum gap length between two connection points. A path will only be connected if the distance between two connection points is less than the maximum gap distance; otherwise, the connection points are considered to be on different paths and no path connection will occur, as shown in Figure S9. Finally, two-dimensional Fourier inverse transformation is used for spatial projection transformation reconstruction of two-dimensional images to achieve spatial three-dimensional reconstruction of electrical tree fluorescence signals.


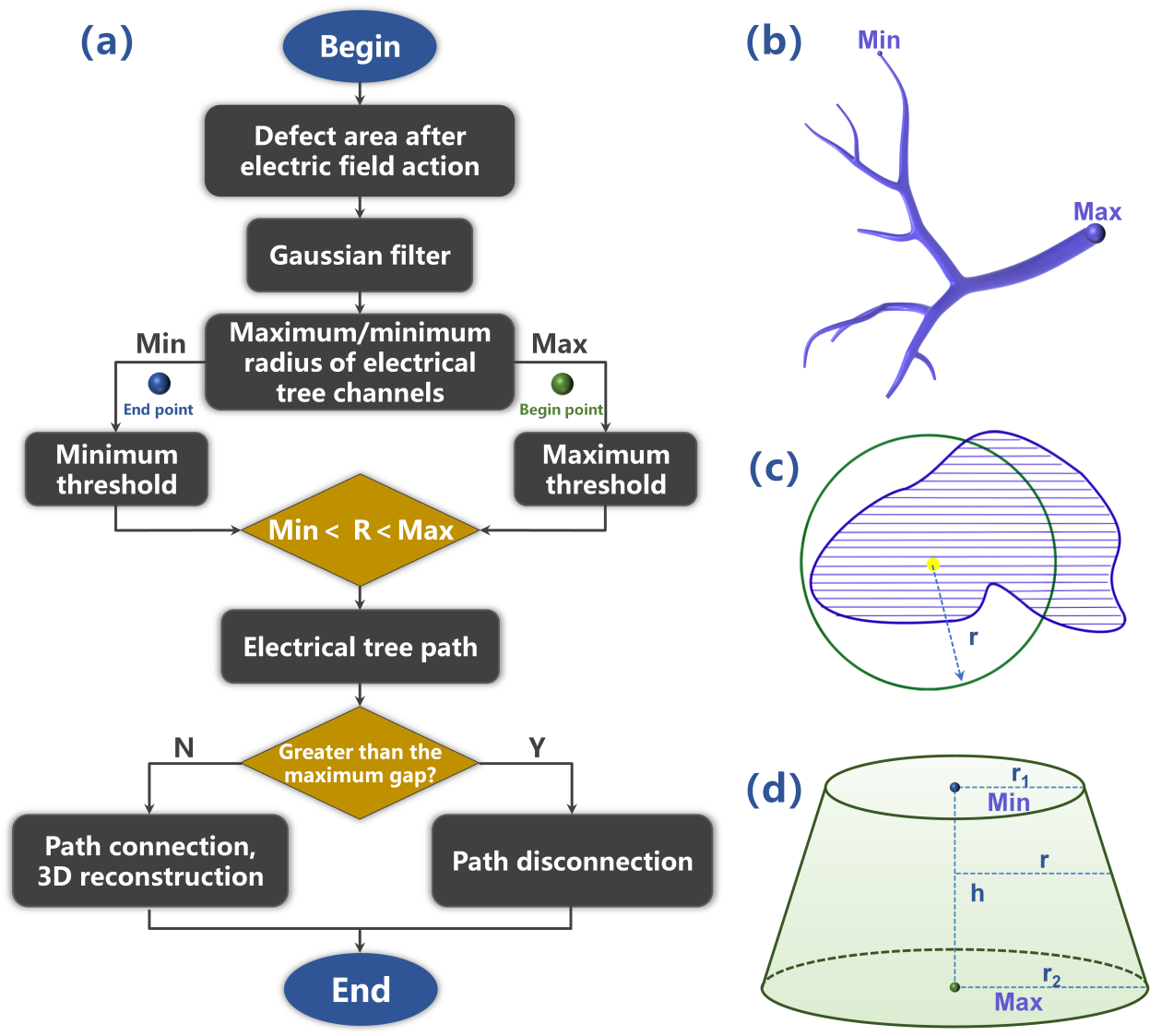


**Figure S9.** Schematic diagram of the 3D morphological reconstruction process for autofluorescence in electrical trees.

1. **Comparison of the original and reconstructed 3D morphologies of electrical tree autofluorescence**

The original 3D imaging results of electrical tree autofluorescence before reconstruction are shown in Figure S10(a), where numerous noise points can be observed within the background signals, which interfere with the geometric analysis of the fluorescent morphology of the electrical trees after imaging. This paper adopts the aforementioned automatic path planning algorithm to perform 3D reconstruction of the fluorescent morphology of the electrical trees. To better illustrate the changes in the morphology of the electrical trees before and after 3D reconstruction, the electrical trees are set to undergo 3D reconstruction along the x, y, and z directions respectively, with the results shown in Figures S10(b-d). The final result after 3D reconstruction is presented in Figure S10(e).


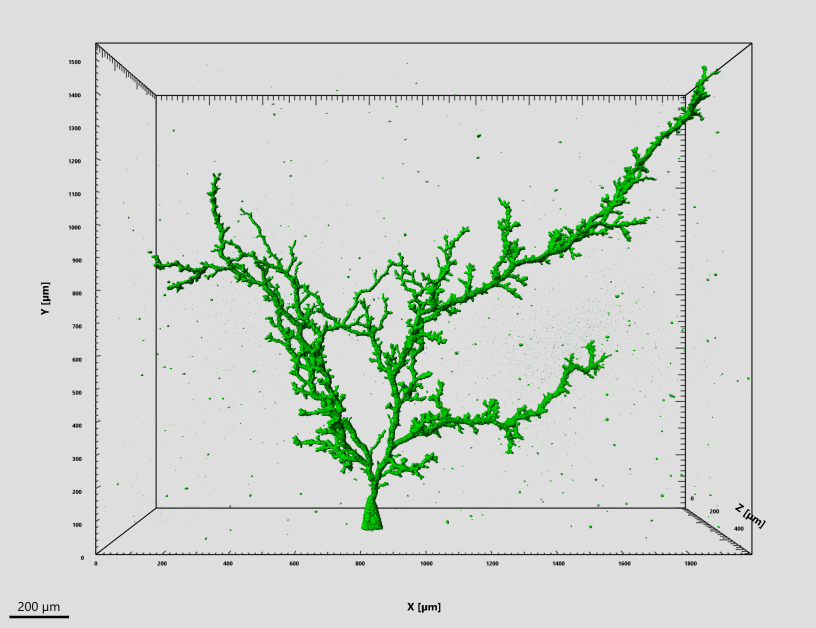


**Figure S10 (a)** The original 3D fluorescent morphology of the electrical trees before 3D reconstruction.

**
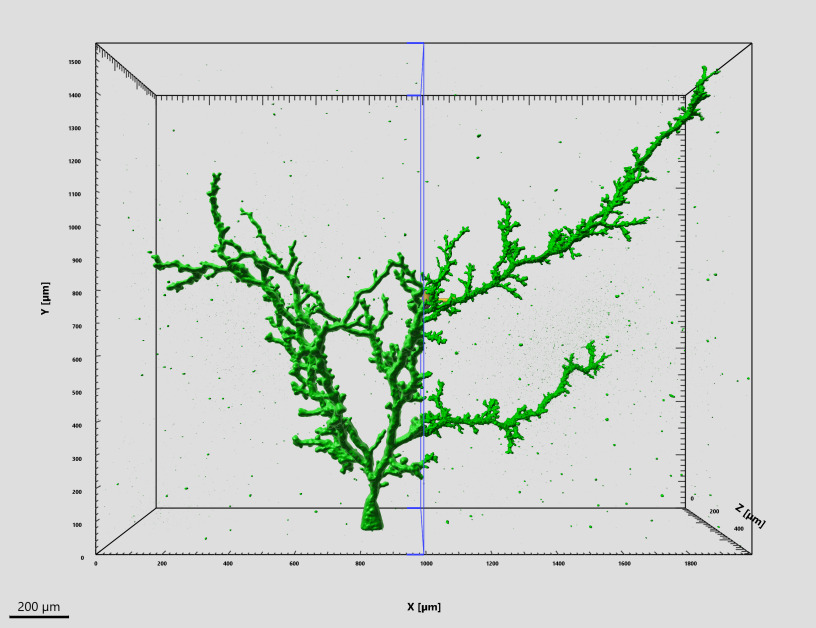
**

**Figure S10 (b)** The process of 3D reconstruction of the electrical trees along the *x* direction, with the reconstructed image on the left side and the original image on the right side.


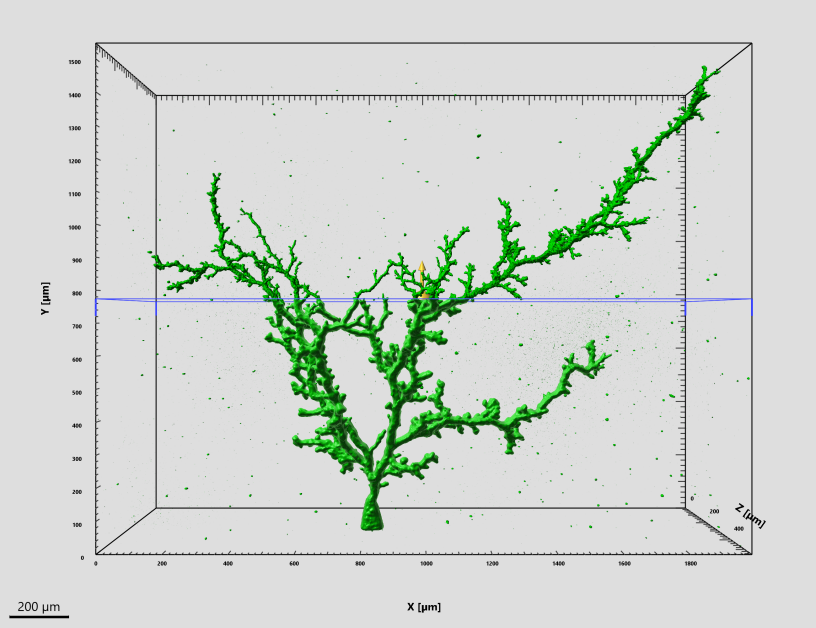


**Figure S10 (c)** The process of 3D reconstruction of the electrical trees along the *y* direction, with the reconstructed image at the bottom and the original image at the top.

**
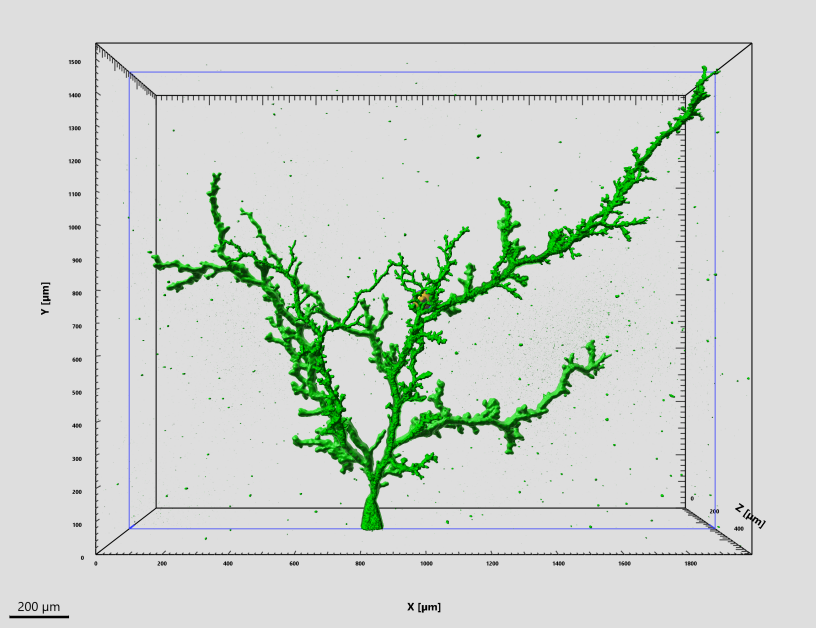
**

**Figure S10 (d)** The process of 3D reconstruction of the electrical trees along the *z* direction, with the reconstructed image at the bottom and the original image at the top.

**
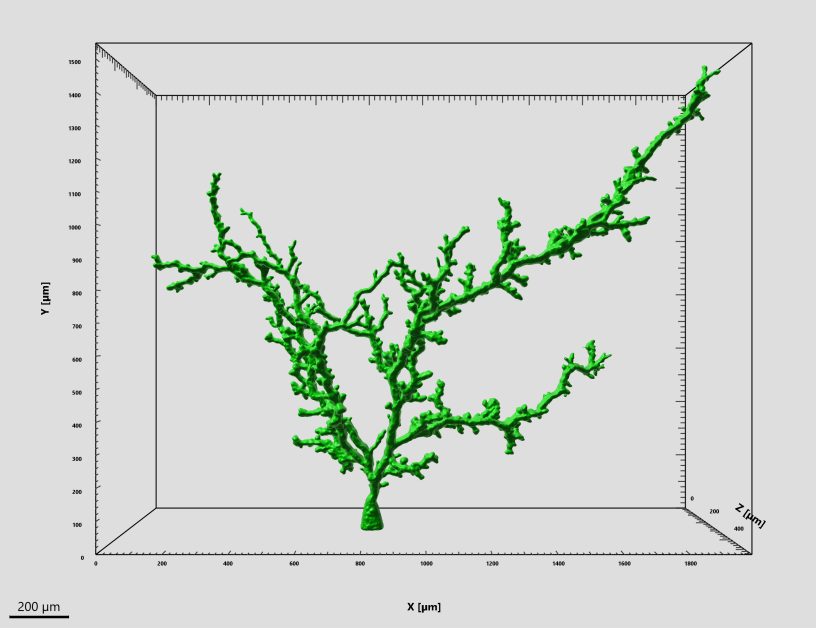
**

**Figure S10 (e)** The final 3D fluorescent morphology of the electrical trees after 3D reconstruction.

1. **The effects of varying needle electrode placement and quantity on electrical tree morphology**

To address the lack of information regarding measurements conducted near the original needle electrode test site, we will investigate the results of reinserting a needle electrode near the initial location (2 mm from the original site) and performing measurements. These findings will be added to the supporting information, with detailed results presented in Figure S11.

(1) Initial Implantation: The first needle electrode was positioned with its tip 2 mm from the ground electrode/epoxy interface (labeled "First needle electrode"). Following the application of 12 kV for 10 minutes, three-dimensional autofluorescence imaging revealed a well-defined electrical tree structure ("The first electrical tree").

(2) Proximal Reinsertion: After removing the first electrode, a second needle was reinserted 2 mm adjacent to the original site (labeled "Second needle electrode"). Under identical voltage exposure conditions, a distinct electrical tree formed, growing predominantly toward the left (labeled "The second electrical tree"). This directional bias is attributed to the combined effects of internal gas pockets and localized electric field distortion induced by the new electrode position.

Notably, autofluorescence imaging consistently resolved three-dimensional defect structures even when probing adjacent locations, demonstrating the technique’s spatial resolution capability. The observed morphological variations between successive measurements highlight the influence of microscopic heterogeneities on electrical tree propagation pathways.

**
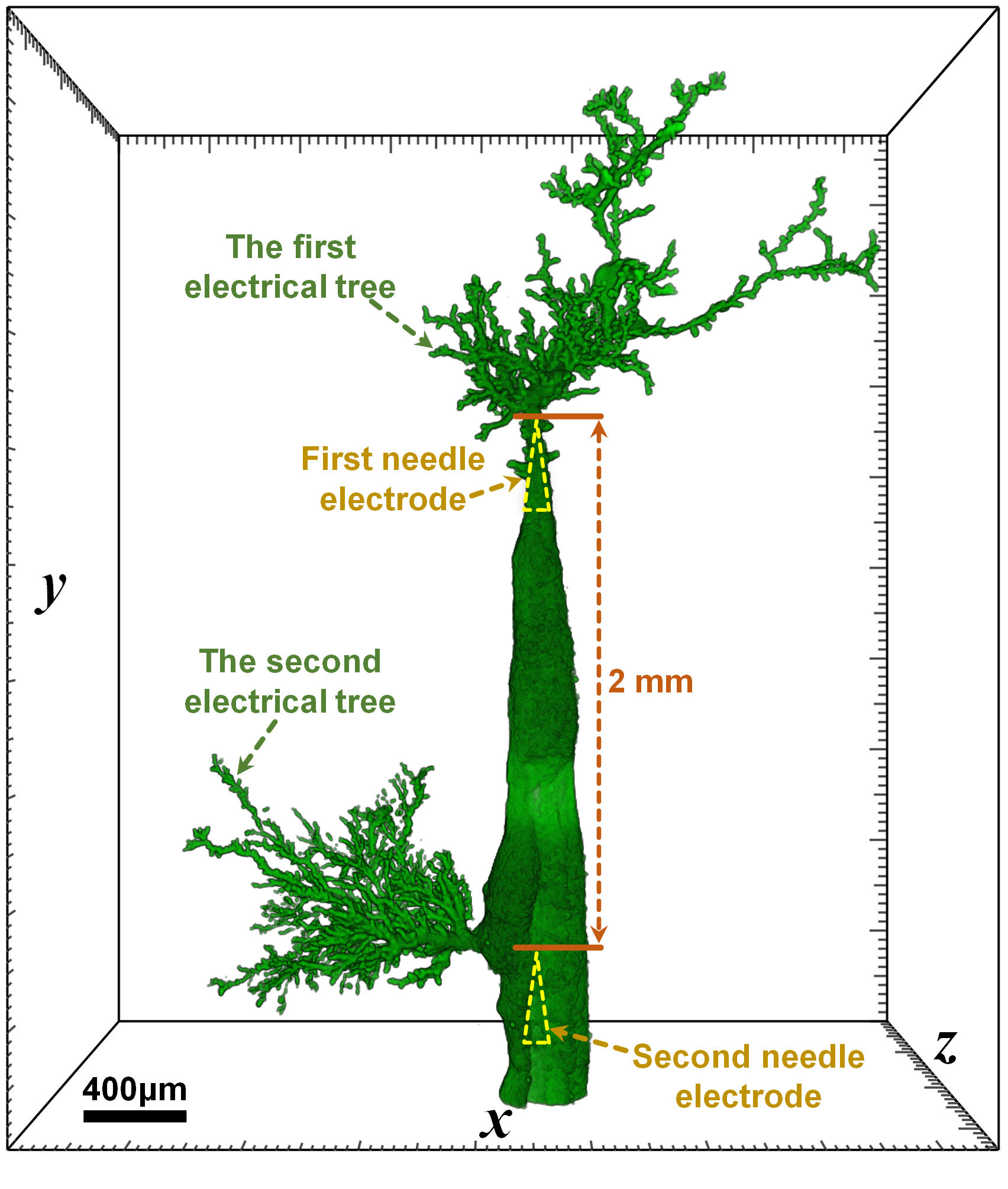
**

**Figure S11.** The effects of varying needle electrode placement (anterior/posterior positions) on electrical tree morphology.

In order to evaluate the effect of using multiple needle electrodes simultaneously on the morphology of electrical trees, we executed supplementary investigations in epoxy resin systems. Our approach involved embedding multiple short needles at non-uniform locations within the epoxy matrix, proximal to its upper surface, during sample synthesis. Subsequent application of 12 kV for 30 minutes, followed by three-dimensional autofluorescence imaging (visualized in Supporting Information Figure S12), demonstrated the successful generation of multiple, well-defined electrical trees. The observed randomness in their spatial arrangement appears driven by a synergy between (i) Localized electric field perturbations originating from the arbitrarily placed needle tips, and (ii) Pre-existing microstructural variability within the epoxy (encompassing voids and impurities) dictating preferential tree growth paths.


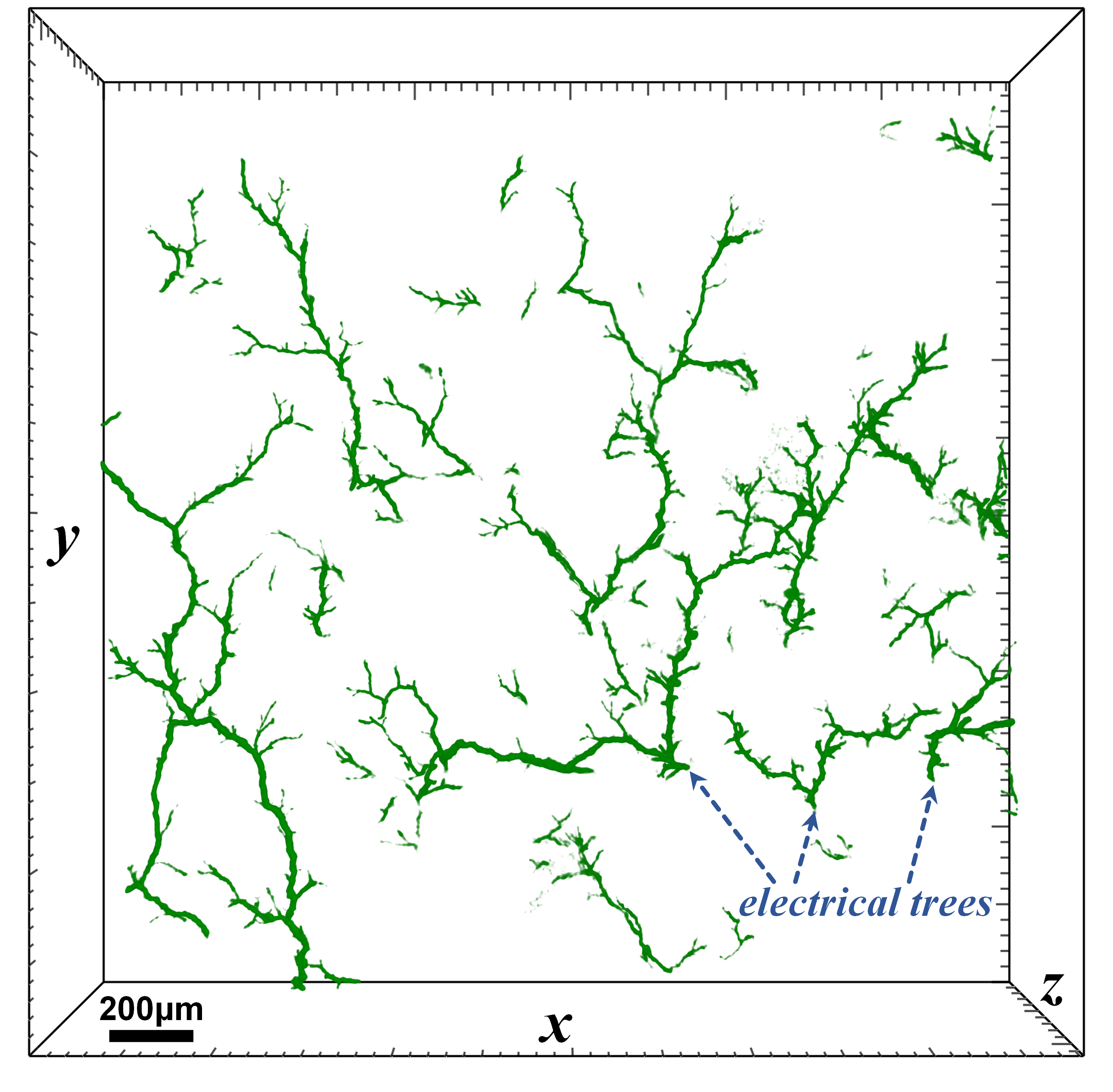


**Figure S12.** The effects of varying needle electrode quantity on electrical tree morphology.

1. **Testing of physicochemical properties in the electrical tree region inside epoxy resin**

**10.1 Raman spectroscopy analysis**

As shown in the Raman spectroscopy analysis results in Figure S13, the main characteristic peaks related to the benzene ring in the epoxy resin are located at 1612 cm⁻¹ and 1116 cm⁻¹. These two peaks correspond to the stretching vibration of C-C bonds, while the peak at 1189 cm⁻¹ is attributed to the in-plane bending vibration of C-H bonds. The characteristic peaks of epoxy groups are mainly concentrated in the range of 1220 cm⁻¹ to 1280 cm⁻¹, which are associated with the vibration of epoxides. With the increase of the voltage application time of 12 kV, the characteristic peaks of epoxy groups gradually weaken, which may be due to the ring-opening reaction of some epoxy groups during the development of electrical tree damage. In addition, the peak near 916 cm⁻¹ is considered to be related to the deformation of the epoxy ring in the epoxy resin. As the voltage application time of 12 kV increases, this peak almost completely disappears, which further confirms that the epoxy groups undergo cracking during the formation of electrical trees. These results indicate that the development of electrical tree damage not only affects the chemical structure of the epoxy resin but also leads to chemical changes in the epoxy groups, thereby altering the Raman spectral characteristics of the material.


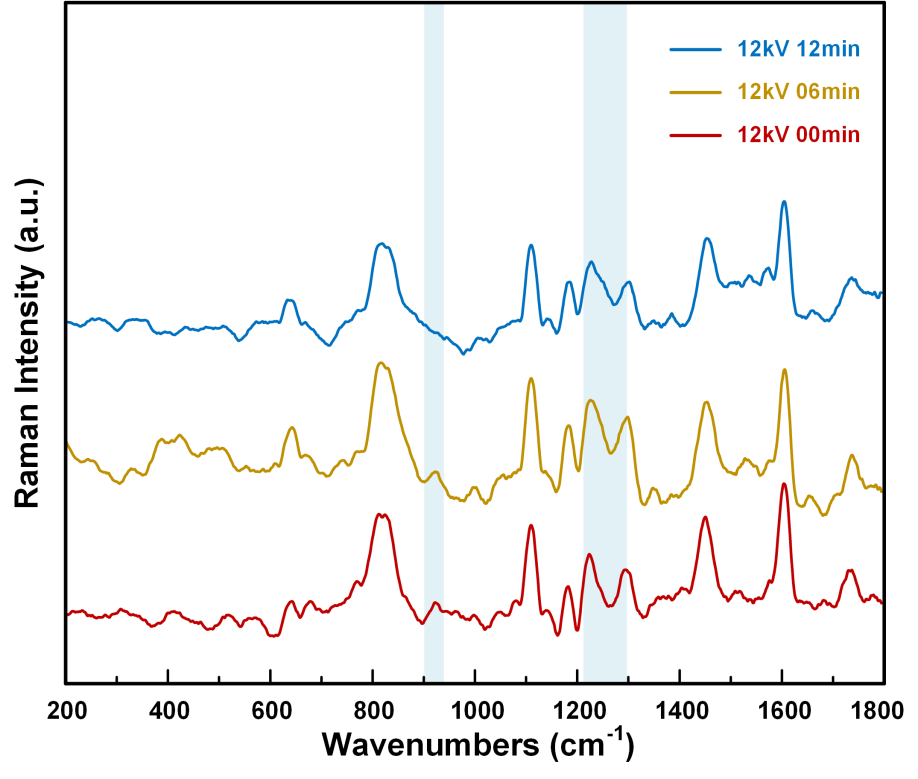


**Figure S13.** Raman spectra of the electrical tree region inside the epoxy resin under different voltage application times of 12 kV.

**10.2 Fourier Transform Infrared (FTIR) Spectroscopy Analysis**

The time interval between the termination of high-voltage application (12 kV) and the initiation of TG-infrared analysis was strictly controlled to within 30 minutes for all samples. Unless otherwise specified, measurements were taken immediately within 3 minutes after the end of pressurization.

As shown in the infrared spectrum in Figure S14, the -OH stretching vibration peak in free H₂O molecules is near 3650 cm⁻¹, and as the voltage application time of 12 kV increases, this peak gradually becomes gentler, which may be because H₂O molecules escape and disappear into the air during the electrical tree damage process. The methyl (-CH₂-) and methine (-CH-) in the epoxy resin exhibit C-H stretching vibration absorption peaks in the range of 2900 cm⁻¹ to 3000 cm⁻¹. The characteristic peaks of aromatic rings in the epoxy resin are at 1712 cm⁻¹ and 1514 cm⁻¹. The symmetric epoxy ring breathing vibration peak is located at about 1288 cm⁻¹, and the asymmetric C-O-C stretching vibration peak is at about 904 cm⁻¹. With the increase of the voltage application time of 12 kV, the characteristic peaks of the epoxy ring gradually disappear, which may be due to the ring-opening degradation of some epoxy rings when electrical tree damage occurs. The absorption peak of ether bond vibration at about 1000 cm⁻¹ and the C-O stretching vibration peak at 1178 cm⁻¹ both gradually disappear as the voltage application time of 12 kV prolongs. This change indicates that the epoxy resin undergoes thermal cracking under the action of an electric field, especially the C-O chemical bond has a greater degree of fracture, and provides direct evidence for the molecular structure change of the epoxy resin during the electrical tree damage process.

Additionally, comparative analysis of the blue curve (12 kV applied for 12 min, tested immediately post-termination) and black curve (12 kV applied for 12 min, stored under ambient conditions for 30 min prior to analysis) in Figure S14 reveals no observable differences in infrared absorption spectra between samples analyzed immediately after voltage termination and those analyzed after a 30-minute ambient storage period.


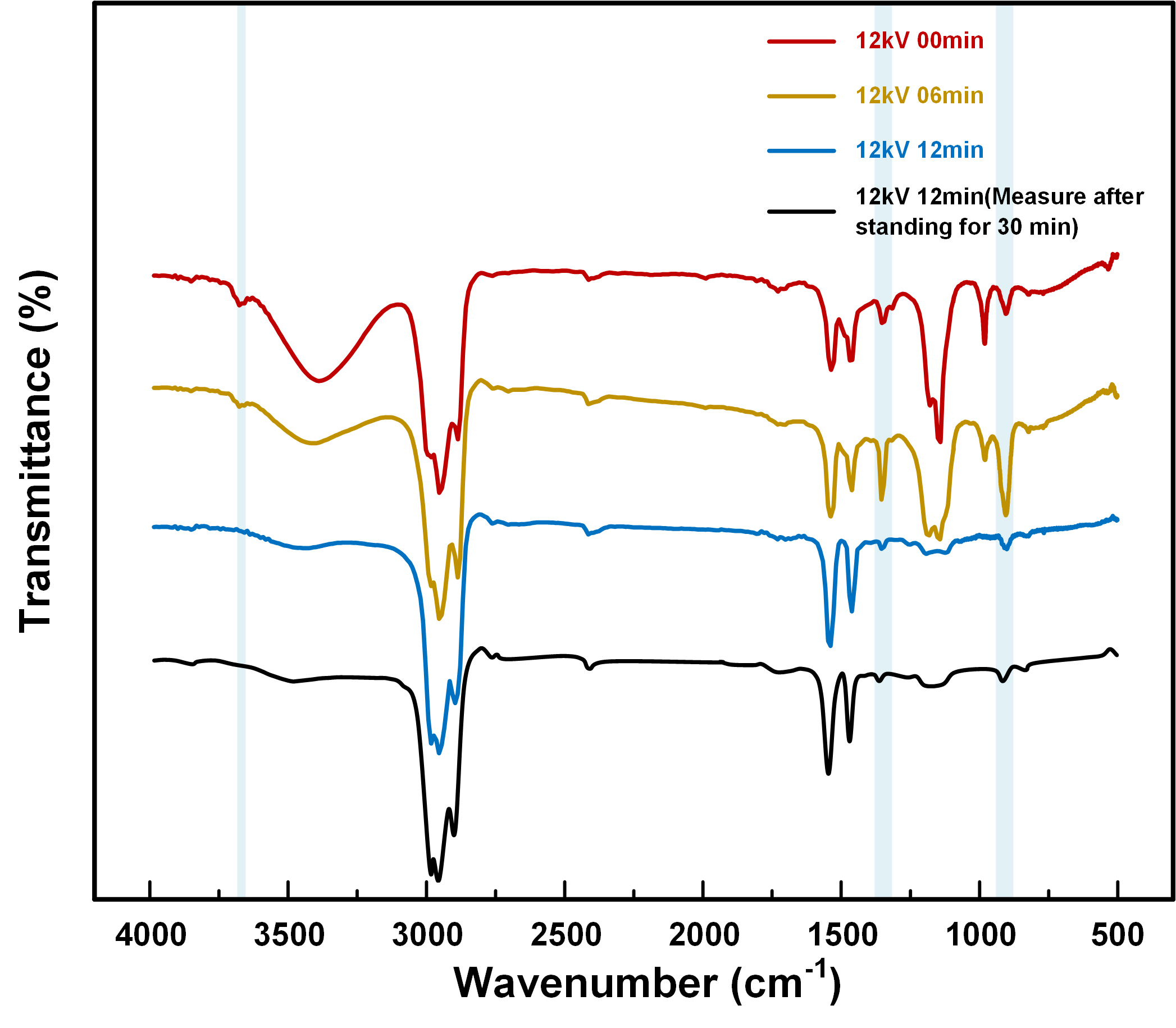


**Figure S14.** FTIR spectra of the electrical tree region inside the epoxy resin under different voltage application times of 12 kV.

**10.3 Thermal Gravimetric (TG) Analysis**

As shown in the thermogravimetric analysis curve in Figure S15, the pure epoxy resin sample without electric field application undergoes thermal weight loss between 300 ℃ and 600 ℃, and this process is represented by the red curve in the TGA curve. With the increase of the voltage application time of 12 kV, the maximum weight loss temperature of the epoxy resin increases from 450 ℃ to 473 ℃, and this change is shown in the TGA curve by the yellow and blue curves. This indicates that the epoxy resin sample with electrical tree channels completes thermal decomposition in a higher temperature range, suggesting that local thermal cracking may have occurred inside the epoxy resin under the action of an electric field, resulting in the residual thermal cracking products requiring further decomposition at higher temperatures.

Similarly, comparative analysis of the blue curve (12 kV applied for 12 min, tested immediately post-termination) and black curve (12 kV applied for 12 min, stored under ambient conditions for 30 min prior to analysis) in Figure S15 demonstrates no detectable discrepancies in thermogravimetric (TG) curves between samples analyzed immediately after voltage termination and those analyzed after a 30-minute ambient storage period.

Preliminary control experiments in Figures S14–S15 collectively confirm that this short time interval—applying 12 kV voltage for 12 minutes, followed by 30 minutes of storage at room temperature prior to testing—does not induce significant changes in the thermal decomposition behavior of the samples.


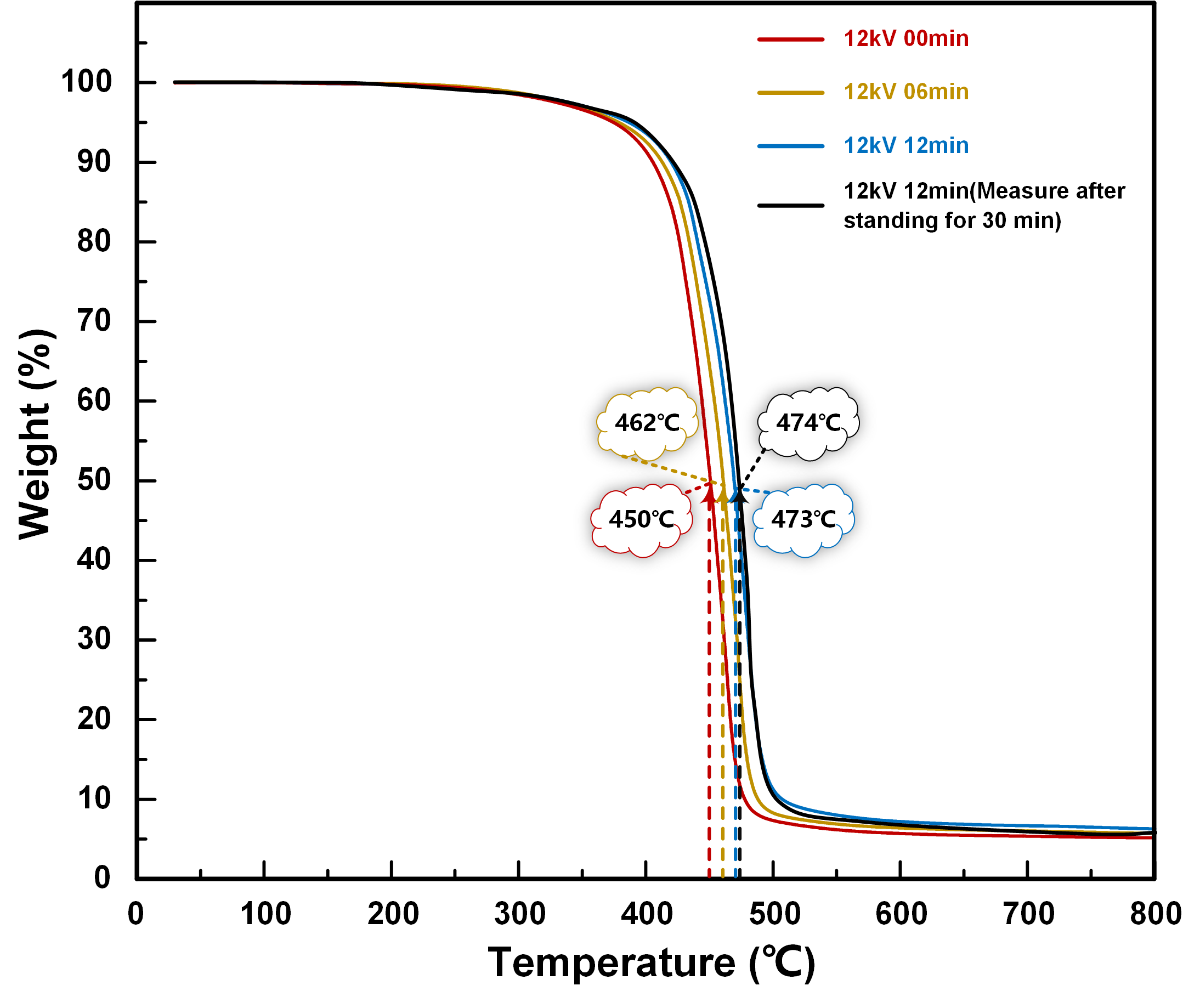


**Figure S15.** Thermal gravimetric curves of the electrical tree region inside the epoxy resin under different voltage application times of 12 kV.

1. **Molecular Dynamics Simulation of Epoxy Resins**

The ReaxFF (Reactive Force Field) serves as an advanced molecular dynamics simulation tool that can model the formation and breaking of chemical bonds during reactions, thereby revealing the reaction mechanisms involved in bond cleavage and formation within polymers under coupled electro-thermal processes. By establishing a ReaxFF molecular dynamics model, we investigated the impact of coupled electro-thermal stresses on the epoxy resin system during the development of electrical trees, thereby elucidating the microscopic degradation mechanisms of the epoxy resin system in this process. In this study, we selected bisphenol A diglycidyl ether (DGEBA) as the epoxy resin matrix and methylhexahydrophthalic anhydride (MHHPA) as the curing agent to construct the epoxy resin system. The general molecular structure formula of DGEBA and the molecular structure of MHHPA are shown in Figure S16.


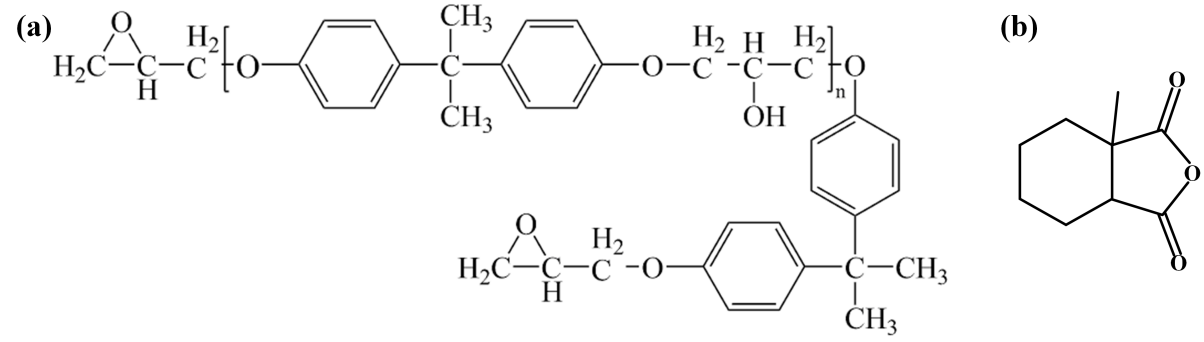


**Figure S16.** (a) General molecular structure formula of DGEBA; (b) General molecular structure of MHHPA.

The epoxy equivalent weight of DGEBA used is approximately 196 (185~208), and it is liquid at room temperature, so the degree of polymerization (n) value is 0 or 1^[23]^. When the proportion of DGEBA molecules with n = 1 is 20%, the total epoxy equivalent weight of the system falls within the raw material range used in this article. When the degree of polymerization (n) values are 0 or 1, respectively, the molecular structures of DGEBA are shown in Figure S17.


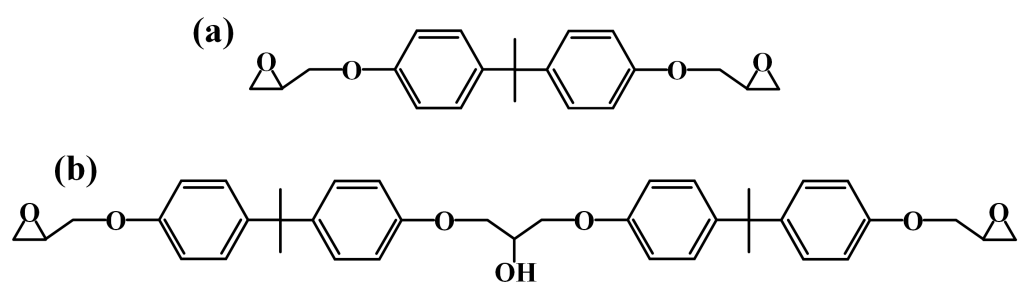


**Figure S17.** Molecular structures of DGEBA with (a) a degree of polymerization (n) value of 0 and (b) a degree of polymerization (n) value of 1.

The principle of the cross-linking reaction between DGEBA and MHHPA: During the reaction, the epoxy group of the DGEBA molecule undergoes a ring-opening reaction with the ester group of the MHHPA molecule. This mainly includes the reaction of the active carbon atom in the epoxy group with the carboxylate anion in the anhydride five-membered ring (Figure S18(a)) and the reaction of the oxygen anion in the epoxy group with the active carbon atom in the anhydride five-membered ring (Figure S18(b)), ultimately forming a cross-linked epoxy resin system.


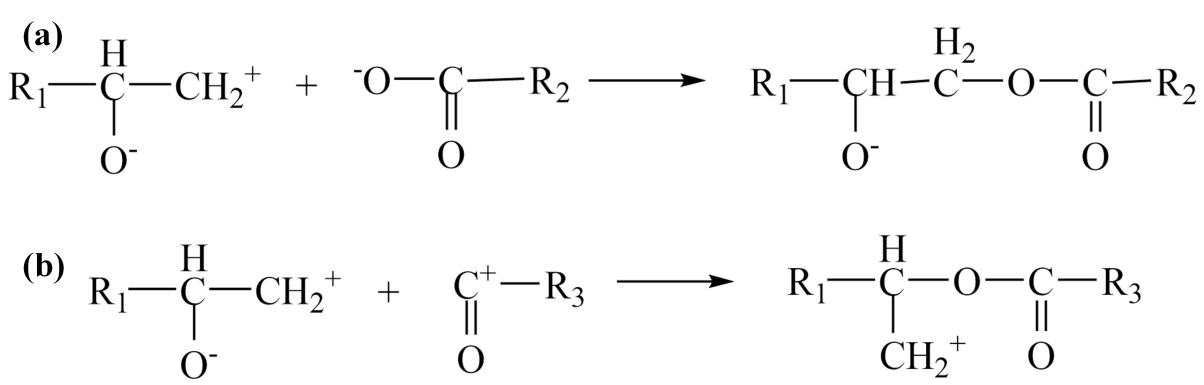


**Figure S18.** (a) Reaction of the active carbon atom in the epoxy group with the carboxylate anion in the anhydride five-membered ring; (b) Reaction of the oxygen anion in the epoxy group with the active carbon atom in the anhydride five-membered ring

During the cross-linking reaction, the epoxy groups in DGEBA molecules with a degree of polymerization (n) of 0 and those with a degree of polymerization (n) of 1 separately undergo a ring-opening reaction with the ester group of the MHHPA molecule. The resulting molecular structure of the epoxy resin after the reaction is shown in Figure S19.


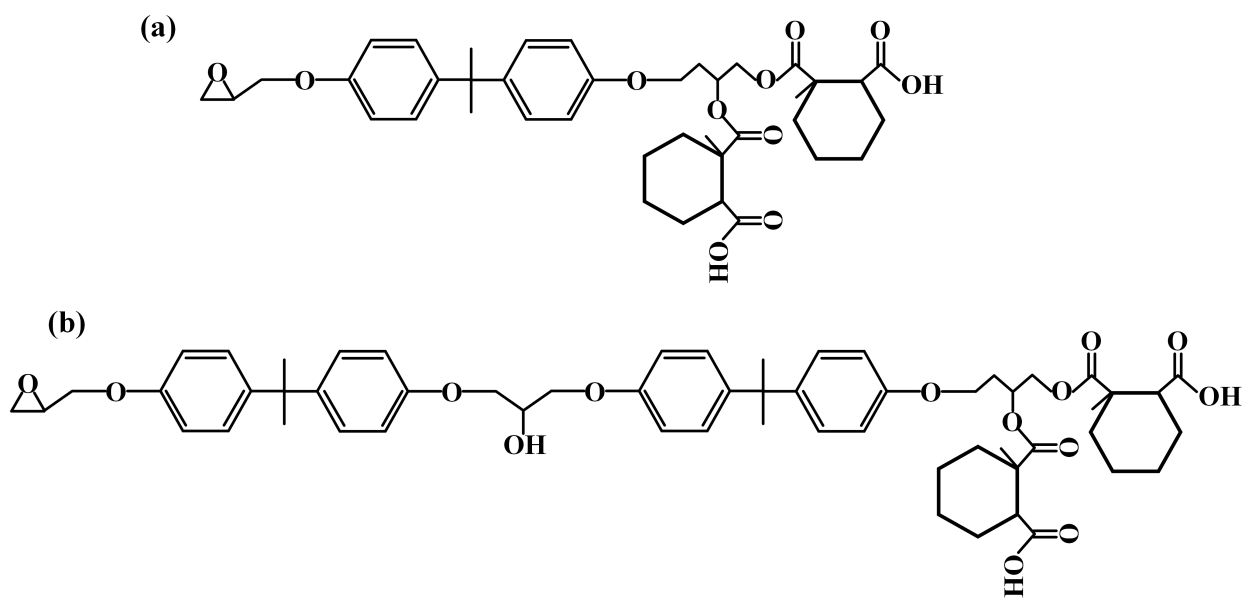


**Figure S19.** (a) Molecular structure of the epoxy resin after cross-linking of DGEBA molecules with a degree of polymerization (n) of 0 and MHHPA molecules; (b) Molecular structure of the epoxy resin after cross-linking of DGEBA molecules with a degree of polymerization (n) of 1 and MHHPA molecules.

Based on a Perl script, the above cross-linking reaction process was simulated, and a molecular dynamics model of the cross-linked epoxy resin system was automatically constructed. The detailed steps are as follows:

(1) Construct molecules of DGEBA with n = 0, DGEBA with n = 1, and MHHPA, totaling three types of molecules. Use the Forcite module to perform geometric optimization on the above molecules, and the optimized monomer molecules are shown in Figure S20(a-c).

(2) Using the Amorphous module, construct an amorphous unit cell with 36 DGEBA molecules with n = 0, 8 DGEBA molecules with n = 1, and 80 MHHPA molecules obtained from step (1), with an initial density value of 0.5 g/cm³. The epoxy resin system before cross-linking is shown in Figure S20(d).

(3) Based on the Perl script and according to the reaction mechanisms shown in Figures S18(a) and (b), simulate the cross-linking and curing process between molecules. Terminate the reaction when the cross-linking degree of the reaction product reaches 95%, and saturate all sites with hydrogen atoms. The initial cross-linked epoxy resin system obtained is shown in Figure S20(e).

(4) For the initial cross-linked system obtained in step (3), perform geometric optimization, annealing, and molecular dynamics simulation in sequence according to the parameter settings shown in Table 1 below. The final configuration is shown in Figure S20(f). The density changes of the cross-linked epoxy resin system before and after molecular dynamics simulation are shown in Figures S20(e) and S20(f), respectively. The average density of the cross-linked and optimized epoxy resin system is 1.086±0.008 g/cm³, which is close to the density of the actual cross-linked and cured epoxy resin (1.1~1.2 g/cm³).

The successful application of ReaxFF in polymer pyrolysis indicates that ReaxFF can reveal the reaction mechanisms of bond cleavage and formation processes in the polymer electro-thermal coupling process we simulated. Since temperature only determines the rate and degree of cracking in ReaxFF molecular dynamics simulations and basically does not affect the reaction path and final reaction products, to shorten the calculation time, thermal cracking simulations were carried out at an environmental temperature of 2000 K for 350 ps, with a pre-equilibration time of 20 ps. The NVT ensemble was used during the simulation, and the time step was set to 0.1 fs. The result of thermal cracking of the cross-linked epoxy resin system under the action of ReaxFF is shown in Figure S20(g).

Table 1. Parameters in geometry optimization, annealing and molecular dynamics simulation

| **Calculation parameters** | **Settings** |
| --- | --- |
| Force field | COMPASS II |
| Charge calculation method | Forcefield assigned |
| Van der Waals force calculation method | Atom based |
| Van der Waals force calculation cutoff radius (Å) | 12.5 |
| Electrostatic force calculation method | Ewald |
| Electrostatic force calculation accuracy (kcal/mol) | 1×10^−3^ |
| Energy convergence criterion (kcal/mol) | 0.001 |


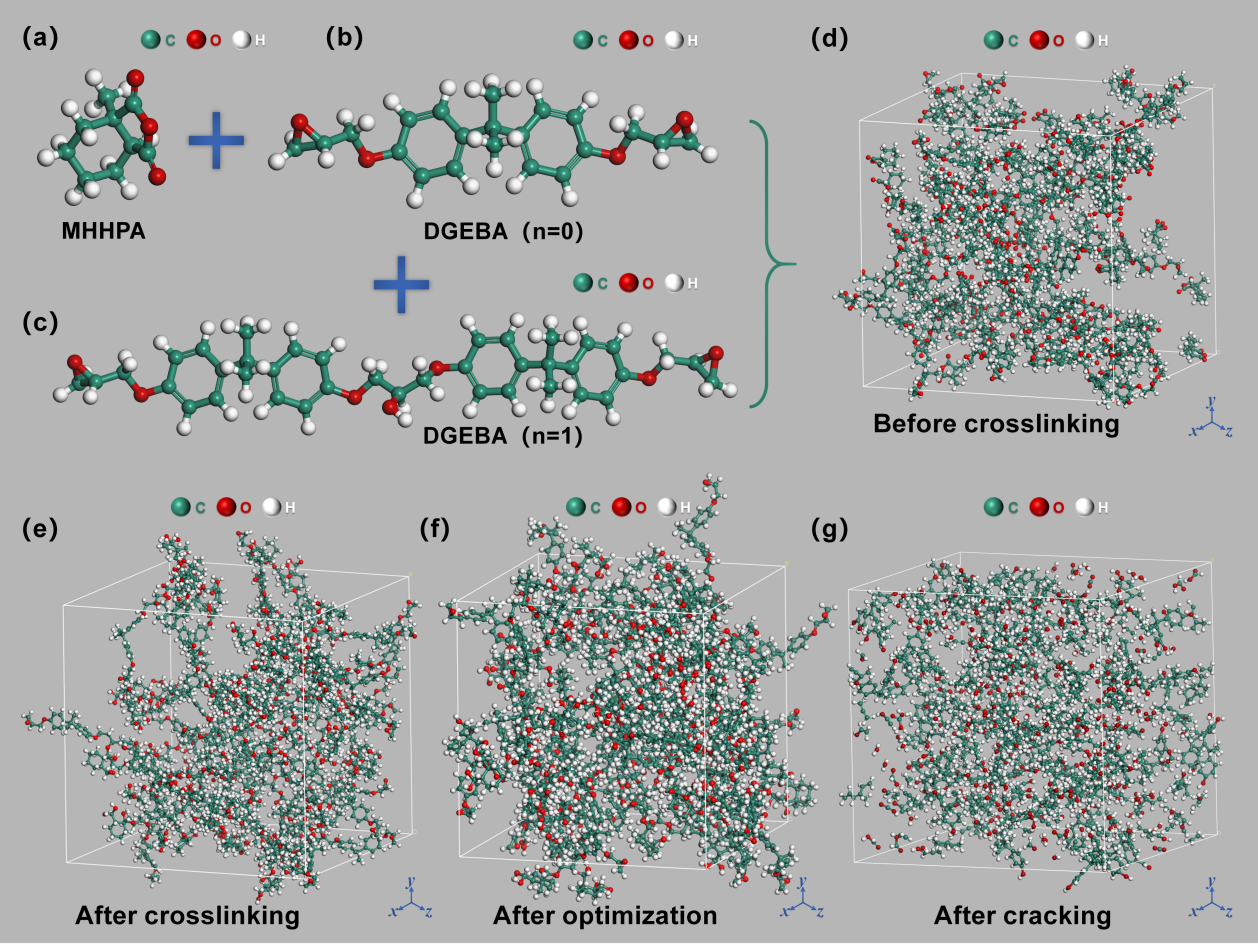


**Figure S20.** Schematic representation of the epoxy resin system before and after crosslinking, as well as before and after thermal cracking.

**References：**

[23] X. M. Yang, Y. Wan, X. Wang, Y. Fu, Z. Y. Huang, Q. Xie, *Compos. Part. B-Eng.* **2019**, *164*, 659-666.

1. **Quantum Chemical Simulation of Epoxy Resins**

The molecular monomers constituting the epoxy resin system before crosslinking are depicted in Figure S21 (a-c) via the GaussView 6.0 interface.


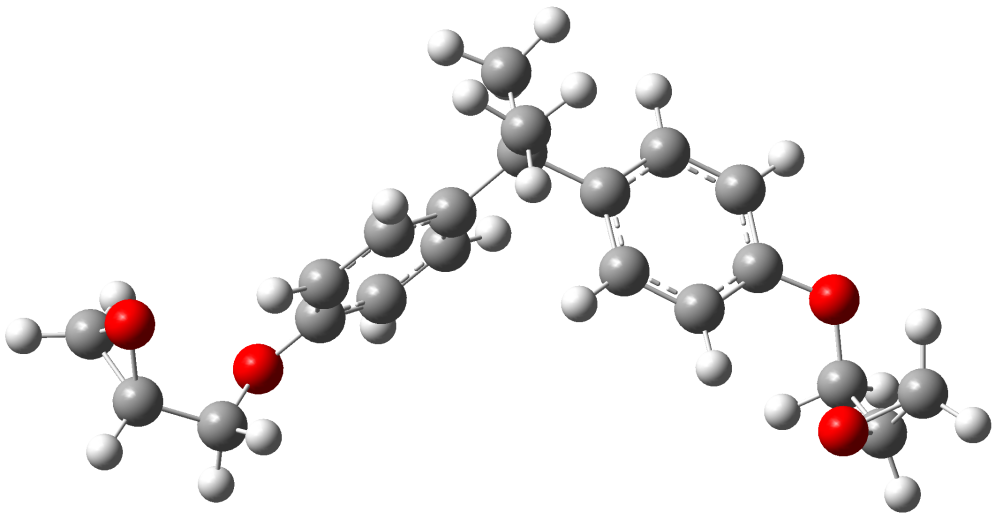


**Figure S21** (a). Molecular structure diagram of DGEBA with a degree of polymerization n=0


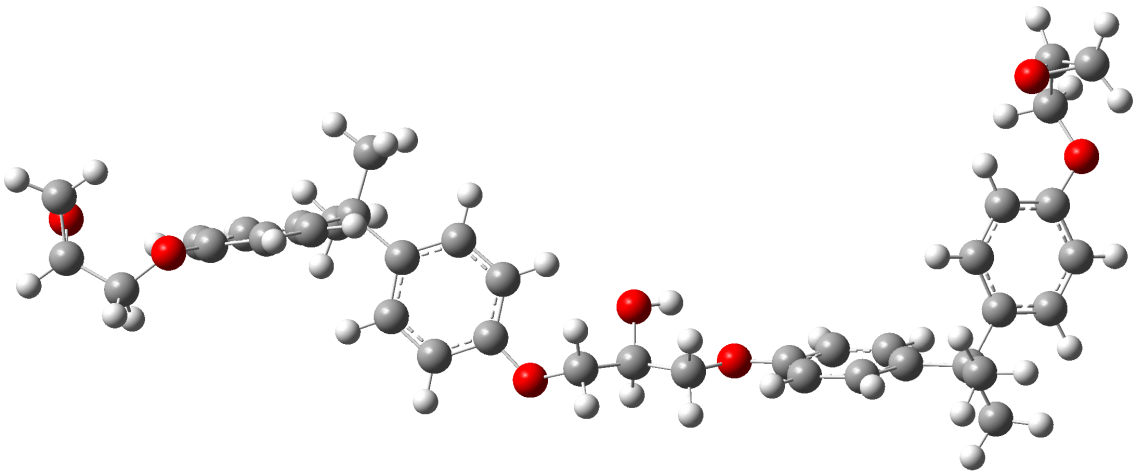


**Figure S21** (b). Molecular structure diagram of DGEBA with a degree of polymerization n=0


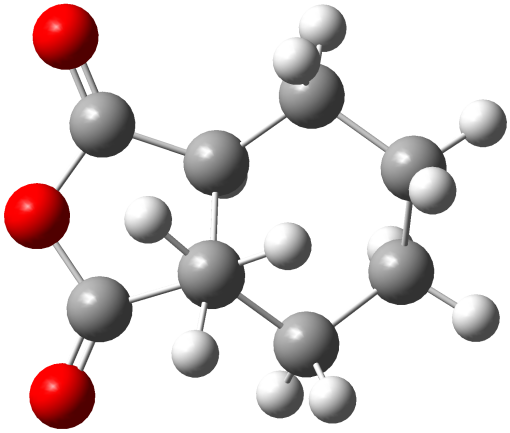


**Figure S21** (c). Molecular structure diagram of MHHPA

The molecular monomers constituting the epoxy resin system after crosslinking are shown in Figure S21 (d-e) through the GaussView 6.0 interface.


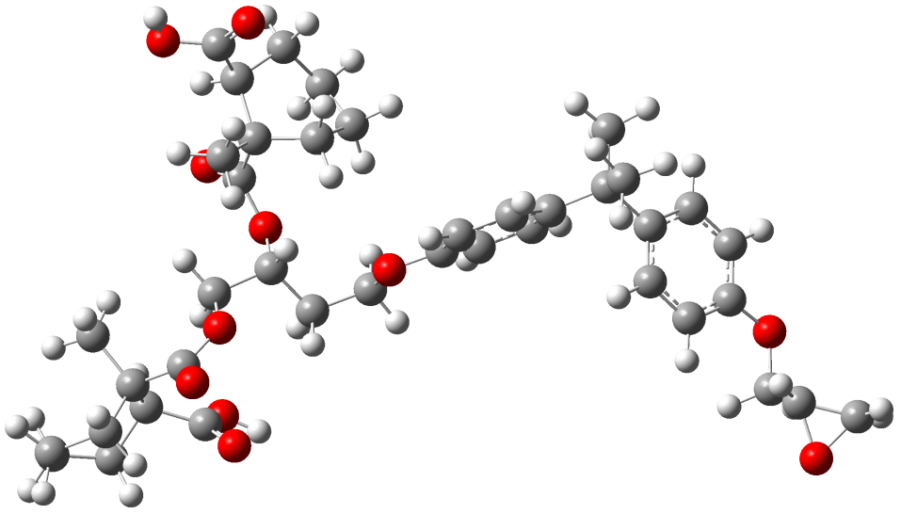


**Figure S21** (d). Molecular structure diagram of the crosslinked epoxy resin formed from DGEBA (n=0) with and MHHPA


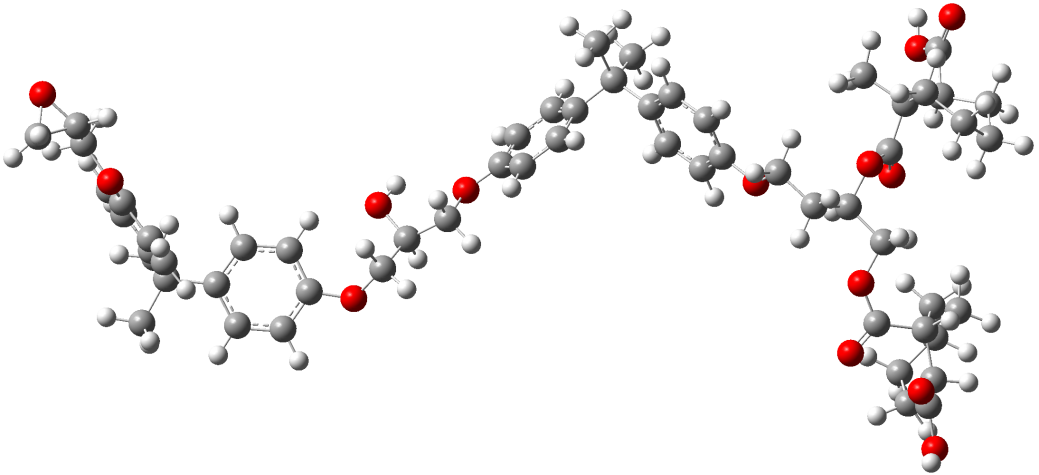


**Figure S21** (e). Molecular structure diagram of the crosslinked epoxy resin formed from DGEBA (n=1) with and MHHPA

Solid organic molecules generated post-thermolysis of the epoxy resin system, such as C_5_H_8_O_3_, C_6_H_8_O, C_8_H_8_, and C_9_H_12_O_2_, were studied using quantum chemical calculations performed with Gaussian 16 B01. Geometric optimization and energy calculations were carried out using the hybrid density functional RB3LYP and the basis set 6-31G(d)^[33,34]^. The structures of some typical solid organic molecules produced after thermal cracking of the epoxy resin system are illustrated in Figure S21 (f-i):


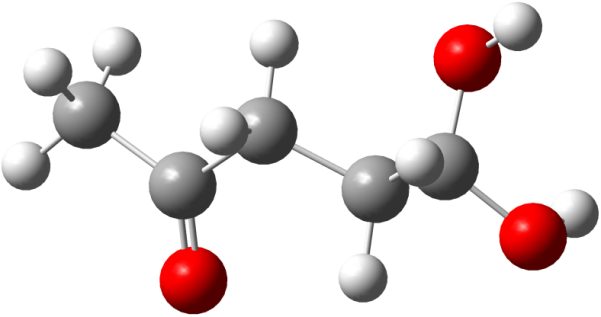


**Figure S21** (f). Molecular structure diagram of C_5_H_8_O_3_


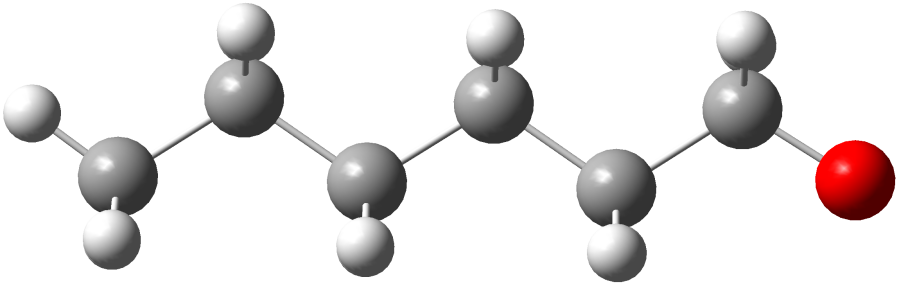


**Figure S21** (g). Molecular structure diagram of C_6_H_8_O


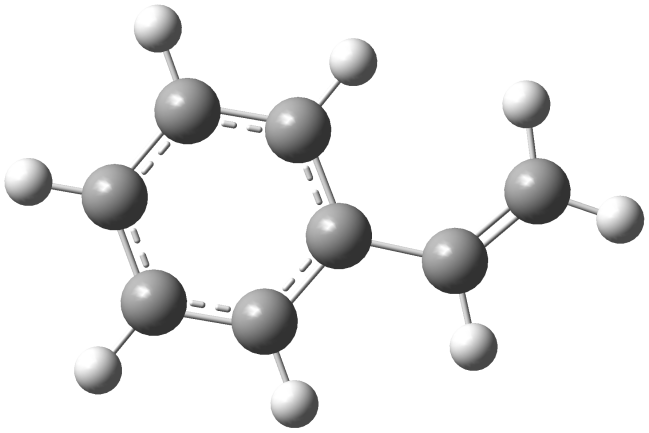


**Figure S21** (h). Molecular structure diagram of C_8_H_8_


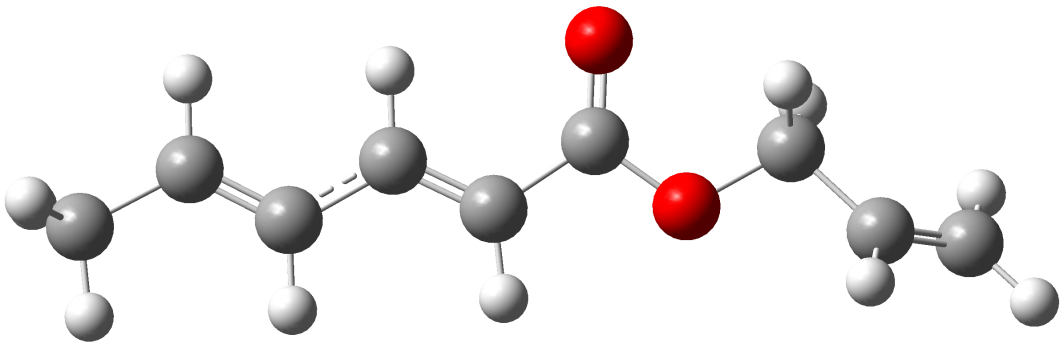


**Figure S21** (i). Molecular structure diagram of C_9_H_12_O_2_

**References：**

[33] T. Lu, Q. X. Chen, *ChemPhysChem* **2021**, *22*, 386-395.

[34] T. Stuyver, D. Danovich, J. Joy, S. Shaik, *Wires. Comput. Mol. Sci*. **2020**, *10*, e1438.

1. **Phase Field Simulation of Electrical Trees within Epoxy Resin**

In Figure S22, we simulated the entire process of electrical tree defects from initiation to final breakdown using a phase field model. During the initial 60 ms, the development of electrical trees was relatively slow, but they rapidly expanded between 90 and 110 ms, leading to eventual breakdown. The dynamic changes in the phase field variable during the development of electrical tree defects are detailed in Figure S22 (a1-a12).


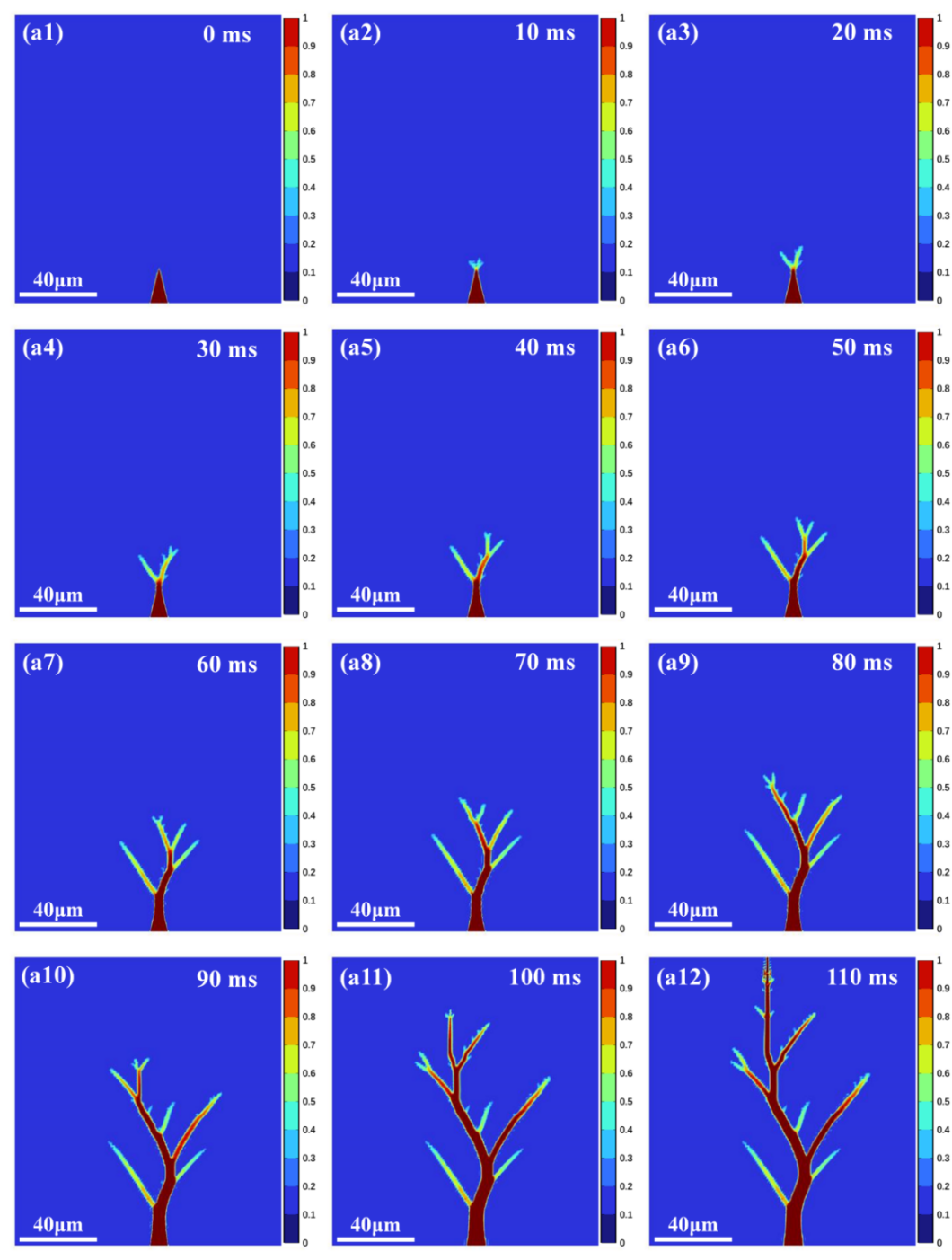


**Figure S22** (a1-a12). Dynamic results of the phase field variable during the development of electrical trees

The dynamic changes in the electric field variable during the development of electrical tree defects are shown in Figure S22 (b1-b12). Real-time changes in both phase field and electric field variables can be seen in Movie 4. These simulation results not only provide insights into predicting the size and morphology of electrical trees during their development but also offer important references for understanding the variations in electric field strength throughout the process.


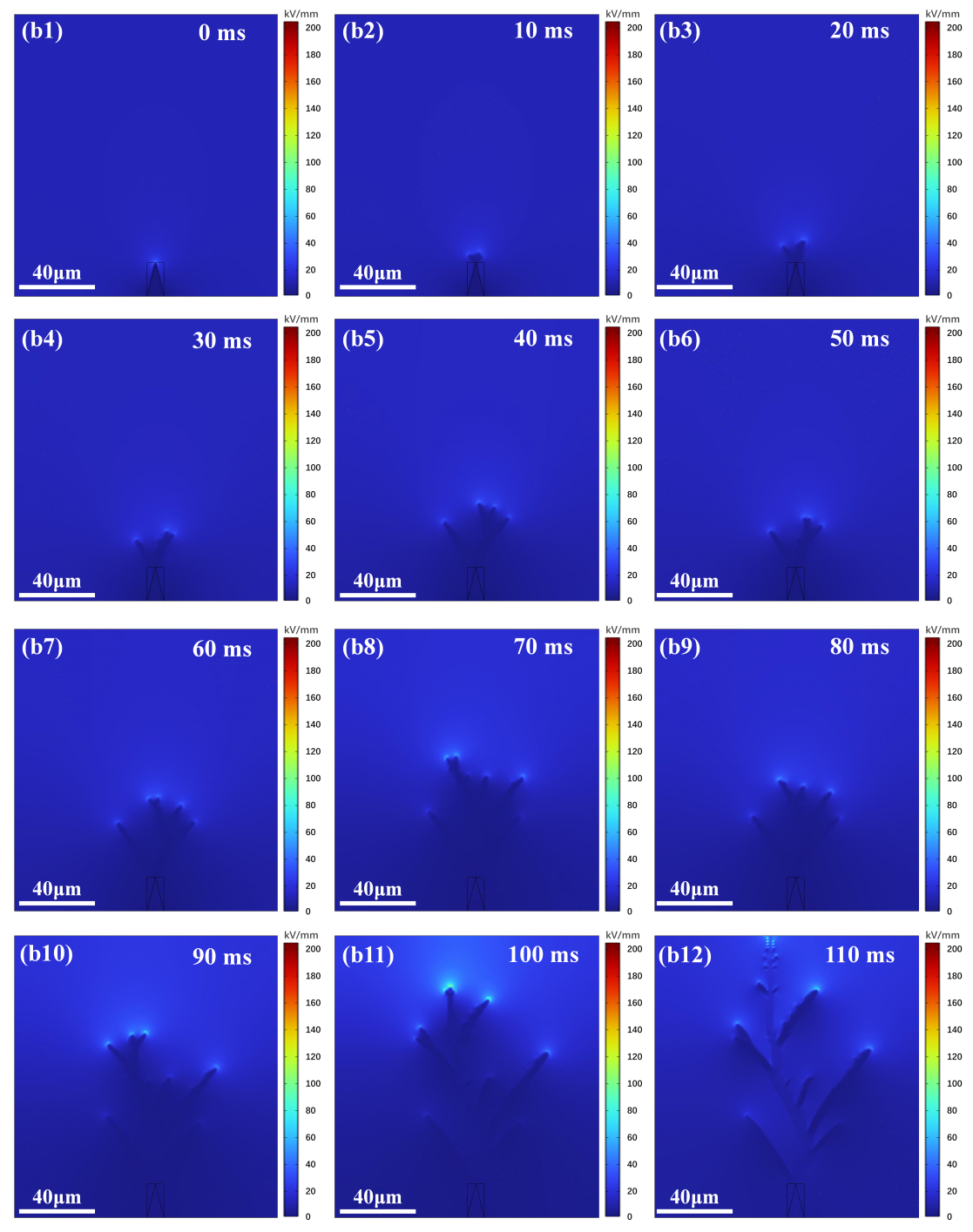


**Figure S22** (b1-b12). Dynamic results of the electric field variable during the development of electrical trees.

The regions where electrical trees develop can be equivalent to areas with high dielectric constants. Under the phase field model, the development of electrical trees can be understood as a phase transition process of the phase field variable related to the dielectric constant under external electric field conditions. For more details on the principles of phase field simulation, see Equations (1)-(5) in the main text.
